# Novel ensemble intelligence methodologies for rockburst assessment in complex and variable environments

**DOI:** 10.1038/s41598-022-05594-0

**Published:** 2022-02-03

**Authors:** Diyuan Li, Zida Liu, Danial Jahed Armaghani, Peng Xiao, Jian Zhou

**Affiliations:** 1 School of Resources and Safety Engineering, Central South University, Changsha, 410083 China; 2 Department of Urban Planning, Engineering Networks and Systems, Institute of Architecture and Construction, South Ural State University, 76, Lenin Prospect, Chelyabinsk, Russia 454080

**Keywords:** Natural hazards, Computer science, Statistics

## Abstract

Rockburst is a severe geological hazard that restricts deep mine operations and tunnel constructions. To overcome the shortcomings of widely used algorithms in rockburst prediction, this study investigates the ensemble trees, i.e., random forest (RF), extremely randomized tree (ET), adaptive boosting machine (AdaBoost), gradient boosting machine, extreme gradient boosting machine (XGBoost), light gradient boosting machine, and category gradient boosting machine, for rockburst estimation based on 314 real rockburst cases. Additionally, Bayesian optimization is utilized to optimize these ensemble trees. To improve performance, three combination strategies, voting, bagging, and stacking, are adopted to combine multiple models according to training accuracy. ET and XGBoost receive the best capabilities (85.71% testing accuracy) in single models, and except for AdaBoost, six ensemble trees have high accuracy and can effectively foretell strong rockburst to prevent large-scale underground disasters. The combination models generated by voting, bagging, and stacking perform better than single models, and the voting 2 model that combines XGBoost, ET, and RF with simple soft voting, is the most outstanding (88.89% testing accuracy). The performed sensitivity analysis confirms that the voting 2 model has better robustness than single models and has remarkable adaptation and superiority when input parameters vary or miss, and it has more power to deal with complex and variable engineering environments. Eventually, the rockburst cases in Sanshandao Gold Mine, China, were investigated, and these data verify the practicability of voting 2 in field rockburst prediction.

## Introduction

Rockburst is a geological calamity often confronted in deep mine operations or deep tunnel excavations, and it has the manners of rock breaking and the sudden release of energy from wall rock^1^. The occurrence of rockburst is generally relevant to lithology, geological structure, surrounding rock mass properties, terrain, and etc. Rockburst, which occurs in many countries^2–5^, is considered a severe danger to the security of employees and equipments in underground construction. Rockburst is a “cancer” in deep mines^6^, killing many South African gold mine employees^7^. With more and more constructions in underground excavations, efficient prediction and prevention of rockburst have become an increasingly crucial topic.

According to Russnes’s method^8^, the rockburst intensity can be classified into four levels (i.e., none, light, moderate and strong). The nature of rockburst is complex and nonlinear, and it is a big challenge to predict rockburst. Numerous technologies have been put forward to evaluate rockburst in the last few decades. These methods include empirical methods, numerical simulation, experimental methods, and intelligent algorithms^3, 9, 10^.

The empirical methods are often applied in the trial implementation phase of underground constructions, including single and multi-index indicators. The single indicators include stress index, energy index, brittleness index, depth index, and so on^3^. The multi-index indicators utilize mathematical methods or other methods to combine the significant factors that are able to control rockburst. The empirical methods are simple and easy to implement. However, they are poorly applicable and only effective in a specific area. Jing et al.^11^ introduced a lot of numerical simulation experiments on rockburst prediction. Wen et al.^12^ applied strain energy density to simulate and investigate the rockburst mechanism. Chen et al.^13^ utilized discontinuity deformation methodology to assess rockburst. Numerical simulation can reveal the failure process of rock^14^. Nevertheless, it is sensitive to input parameters and hard to simulate the dynamic behavior of rockburst. Moreover, the constitutive model in the numerical simulation may not demonstrate the real propriety of the rock. Gong et al.^15^ researched the rockburst tendency of red sandstone by rock experiments. He et al.^16^ adopted indoor experimental methods to study and classify rockburst. Rock mechanics experiments can give some essential information, which is beneficial to study rock properties^17^. However, they are challenging to reproduce real engineering environment and are limited by monitoring and measurement techniques. The empirical method has a narrow scope of application^18^. Numerical simulation for rockburst prediction has high requirements on simulation methods, mechanical constitutive model, and rockburst mechanism^10^. The rock mechanics test for evaluating rockburst requires samples preparation and adequate types of equipment^19^, which is expensive and time consuming. In contrast, the intelligent algorithm is low cost, only focuses on input and output parameters, and has wider applicability^4^. The intelligent algorithm is more worthy for rockburst prediction efficiently and timely with the growing development of big data and artificial intelligence.

Since Feng et al.^20^ utilized neural networks to predict rockburst, many intelligent algorithms have been applied. Table 1 summarizes intelligent algorithms for rockburst prediction in recent years. Each intelligent algorithm has its advantages for specific problems. However, any of these algorithms cannot be perfectly performed in all problems according to the ‘No Free Lunch theory’. There are inevitably some disadvantages in each intelligent algorithm when applied in practical engineering. The discriminant analysis^21^ and logistic regression^22^ are simple and easy to interpret. However, they cannot be applied to complex problems and high-dimensional data. Decision trees^23–25^ can be used for data with missing values, but they tend to overfit. Support vector machine^23, 26, 27^ has a solid theoretical basis, and it is not easy to overfit. However, it performs poorly in multiple classification problems^28^. The *k*-nearest neighbor is efficient and straightforward^23, 29^, but it is sensitive to irrelevant features. Bayes model^23, 30^ is simple and fast in the calculation. However, it requires that features are independent distribution, which is difficult to satisfy in practice. Although neural networks^23, 29, 31, 32^ can deal with more complex problems, they have many hyperparameters to be turned^33^.

**Table 1:** Intelligent algorithms for predicting rockburst in recent years.

Algorithm/model	Input parameters	Data size
LDA^23^	$H,\sigma_{\theta } ,\sigma_{{\text{c}}} ,\sigma_{{\text{t}}} ,\sigma_{\theta } /\sigma_{{\text{c}}} ,\sigma_{{\text{c}}} /\sigma_{{\text{t}}} ,W_{{{\text{et}}}}$	246
QDA^23^	$H,\sigma_{\theta } ,\sigma_{{\text{c}}} ,\sigma_{{\text{t}}} ,\sigma_{\theta } /\sigma_{{\text{c}}} ,\sigma_{{\text{c}}} /\sigma_{{\text{t}}} ,W_{{{\text{et}}}}$	246
PLDA^23^	$H,\sigma_{\theta } ,\sigma_{{\text{c}}} ,\sigma_{{\text{t}}} ,\sigma_{\theta } /\sigma_{{\text{c}}} ,\sigma_{{\text{c}}} /\sigma_{{\text{t}}} ,W_{{{\text{et}}}}$	246
LR^22^	$H,\sigma_{\theta } ,\sigma_{{\text{c}}} ,\sigma_{{\text{t}}} ,W_{{{\text{et}}}}$	135
DT^23^	$H,\sigma_{\theta } ,\sigma_{{\text{c}}} ,\sigma_{{\text{t}}} ,\sigma_{\theta } /\sigma_{{\text{c}}} ,\sigma_{{\text{c}}} /\sigma_{{\text{t}}} ,W_{{{\text{et}}}}$	246
C5.0 DT^25^	$\sigma_{\theta } /\sigma_{{\text{c}}} ,\sigma_{{\text{c}}} /\sigma_{{\text{t}}} ,W_{{{\text{et}}}}$	174
DT^24^	$\sigma_{\theta } /\sigma_{{\text{c}}} ,\sigma_{{\text{c}}} /\sigma_{{\text{t}}} ,W_{{{\text{et}}}}$	132
KNN^23^	$H,\sigma_{\theta } ,\sigma_{{\text{c}}} ,\sigma_{{\text{t}}} ,\sigma_{\theta } /\sigma_{{\text{c}}} ,\sigma_{{\text{c}}} /\sigma_{{\text{t}}} ,W_{{{\text{et}}}}$	246
Naive Bayes^23^	$H,\sigma_{\theta } ,\sigma_{{\text{c}}} ,\sigma_{{\text{t}}} ,\sigma_{\theta } /\sigma_{{\text{c}}} ,\sigma_{{\text{c}}} /\sigma_{{\text{t}}} ,W_{{{\text{et}}}}$	246
Bayesian network^30^	$H,\sigma_{\theta } ,\sigma_{{\text{c}}} ,\sigma_{{\text{t}}} ,W_{{{\text{et}}}}$	135
ANN^23^	$H,\sigma_{\theta } ,\sigma_{{\text{c}}} ,\sigma_{{\text{t}}} ,\sigma_{\theta } /\sigma_{{\text{c}}} ,\sigma_{{\text{c}}} /\sigma_{{\text{t}}} ,W_{{{\text{et}}}}$	246
FA-ANN^31^	$\sigma_{\theta } ,\sigma_{{\text{c}}} ,\sigma_{{\text{t}}} ,\sigma_{\theta } /\sigma_{{\text{c}}} ,\sigma_{{\text{c}}} /\sigma_{{\text{t}}} ,W_{{{\text{et}}}}$	196
ABC-ANN^34^	$\sigma_{\theta } ,\sigma_{{\text{c}}} ,\sigma_{{\text{t}}} ,\sigma_{\theta } /\sigma_{{\text{c}}} ,\sigma_{{\text{c}}} /\sigma_{{\text{t}}} ,W_{{{\text{et}}}}$	246
SVM^23^	$H,\sigma_{\theta } ,\sigma_{{\text{c}}} ,\sigma_{{\text{t}}} ,\sigma_{\theta } /\sigma_{{\text{c}}} ,\sigma_{{\text{c}}} /\sigma_{{\text{t}}} ,W_{{{\text{et}}}}$	246
SVM^26^	$\sigma_{\theta } ,\sigma_{{\text{c}}} ,\sigma_{{\text{t}}} ,\sigma_{\theta } /\sigma_{{\text{c}}} ,\sigma_{{\text{c}}} /\sigma_{{\text{t}}} ,\frac{{\sigma_{c} - \sigma_{t} }}{{\sigma_{c} + \sigma_{t} }},W_{{{\text{et}}}}$	246
SVM^27^	$\sigma_{\theta } ,\sigma_{{\text{c}}} ,\sigma_{{\text{t}}} ,W_{{{\text{et}}}}$	132
GBM^23^	$H,\sigma_{\theta } ,\sigma_{{\text{c}}} ,\sigma_{{\text{t}}} ,\sigma_{\theta } /\sigma_{{\text{c}}} ,\sigma_{{\text{c}}} /\sigma_{{\text{t}}} ,W_{{{\text{et}}}}$	246
RF^23^	$H,\sigma_{\theta } ,\sigma_{{\text{c}}} ,\sigma_{{\text{t}}} ,\sigma_{\theta } /\sigma_{{\text{c}}} ,\sigma_{{\text{c}}} /\sigma_{{\text{t}}} ,W_{{{\text{et}}}}$	246
RF^35^	$\sigma_{\theta } ,\sigma_{{\text{c}}} ,\sigma_{{\text{t}}} ,\sigma_{\theta } /\sigma_{{\text{c}}} ,\sigma_{{\text{c}}} /\sigma_{{\text{t}}} ,W_{{{\text{et}}}}$	246
Voting (BPNN,SVM,DT,KNN,LR,MLR, Naive Bayes)^36^	$H,\sigma_{\theta } ,\sigma_{{\text{c}}} ,\sigma_{{\text{t}}} ,W_{{{\text{et}}}}$	188
Bagging^37^	$\sigma_{\theta } ,\sigma_{{\text{c}}} ,\sigma_{{\text{t}}} ,\sigma_{\theta } /\sigma_{{\text{c}}} ,\sigma_{{\text{c}}} /\sigma_{{\text{t}}} ,W_{{{\text{et}}}}$	102
Boosting^37^	$\sigma_{\theta } ,\sigma_{{\text{c}}} ,\sigma_{{\text{t}}} ,\sigma_{\theta } /\sigma_{{\text{c}}} ,\sigma_{{\text{c}}} /\sigma_{{\text{t}}} ,W_{{{\text{et}}}}$	102
GA-XGB^38^	$\sigma_{\theta } ,\sigma_{{\text{c}}} ,\sigma_{{\text{t}}} ,\sigma_{\theta } /\sigma_{{\text{c}}} ,\sigma_{{\text{c}}} /\sigma_{{\text{t}}} ,W_{{{\text{et}}}}$	275
Stacking(KNN,SVM,DNN,RNN)^29^	$\sigma_{\theta } ,\sigma_{{\text{c}}} ,\sigma_{{\text{t}}} ,\sigma_{\theta } /\sigma_{{\text{c}}} ,\sigma_{{\text{c}}} /\sigma_{{\text{t}}} ,\frac{{\sigma_{c} - \sigma_{t} }}{{\sigma_{c} + \sigma_{t} }},W_{{{\text{et}}}}$	246

LDA = linear discriminant analysis; $H$ = depth; $\sigma_{\theta }$ = maximum tangential stress; $\sigma_{{\text{c}}}$ = uniaxial compressive strength; $\sigma_{{\text{t}}}$ = uniaxial tensile strength; $W_{{{\text{et}}}}$ = the elastic strain index; QDA = quadratic discriminant analysis; PLDA = partial least-squares discriminant analysis; LR = logistic regression; DT = decision tree; KNN = *k*-nearest neighbor; ANN = artificial neural network; FA = firefly algorithm; ABC = artificial bee colony; SVM = support vector machine; BPNN = back propagation neural network; MLR = multiple linear regression; GA = genetical algorithm; XGB = extreme gradient boosting; DNN = deep neural network; RNN = recurrent neural network.

The single model has low robustness, cannot get the optimal solution for all problems, and its performance changes with the variation of engineering environment or input parameters. Accordingly, scholars have attempted to adopt ensemble models to combine multiple models to overcome the shortcomings of a single model. Nonetheless, there are only a few studies in the area of rockburst. Moreover, there is no detailed research on the selection and application of ensemble models in rockburst prediction. To fill the gaps, the present study considers seven models based on decision trees and three combination strategies for rockburst estimation in complex and variable engineering conditions. The seven models include random forest (RF), extremely randomized tree (ET), adaptive boosting machine (AdaBoost), gradient boosting machine (GBM), extreme gradient boosting machine (XGBoost), light gradient boosting machine (LighGBM), and category gradient boosting machine (CatBoost), which all adopt decision trees (DTs) as the basic classifier due to the low bias and high variance of DTs^39^. Three combination strategies are voting, bagging and stacking. These seven models have good performance in machine learning (ML) tasks, but there are no detailed investigations of applying them to rockburst. Furthermore, applying combination strategies to combine multiple single models can make the rockburst models more robust and powerful. Apart from that, Bayesian optimization is implemented to optimize these models. It is significant to note that Bayesian as a highly efficient optimization model, has been widely used in hyperparameter optimization of ML area^40^.

The rest of this study is organized as follows: “Methodology” section describes the techniques and the data from real cases for simulation. Section “Simulation” presents the model parameter optimization and combination, which exhibits the process of selection and integration of the base classifier in detail. In “Results and discussion” section, all models are evaluated to select an optimal model. Moreover, the selected model conducts the sensitivity analysis and is tested for engineering practicability.

## Methodology

### Ensemble trees

#### Random forest and extremely randomized tree

**Figure 1:**
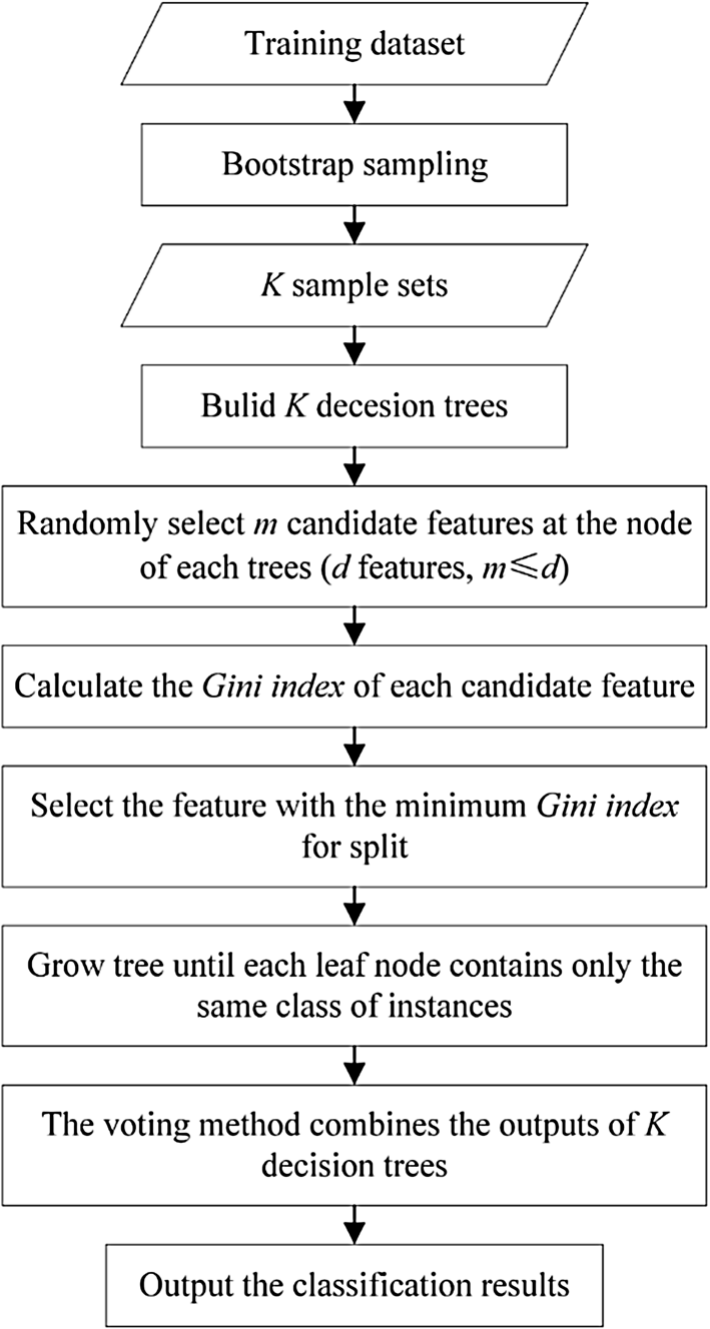
The flowchart to build RF.

RF is the ML model composed of $K$ decision trees. The process to construct the RF is shown in Fig. 1. ET is similar to RF, and the main differences between them are as follows: first, RF uses bootstrap sampling to build a random sample subset, while ET utilizes all original samples, which can reduce the deviation. Secondly, the choice of the split point is different. The RF selects the optimal split point, while the ET randomly chooses the split point, which can reduce the variance. The choice of random split point adds more randomness to the model and speeds up the calculation speed.

1 $$Gini(D) = 1 - \sum\limits_{k = 1}^{|y|} {p_{k}^{2} }$$

2 $$Gini\_index(D,a) = \sum\limits_{V = 1}^{V} {\frac{{\left| {D^{V} } \right|}}{\left| D \right|}} Gini(D^{V} )$$

In Eqs. 1 and 2, $D$ represents the dataset, $\left| y \right|$ is the class number, $p$ is the proportion of each class to the total dataset, $Gini(D^{V} )$ is the *Gini* value of the class $V$, $\left| D \right|$ represents the number of instances, $\left| {D^{V} } \right|$ represents the number of instances of the class $V$, and $a$ represents the feature that needs to be divided.

#### Boosting model

**Figure 2:**
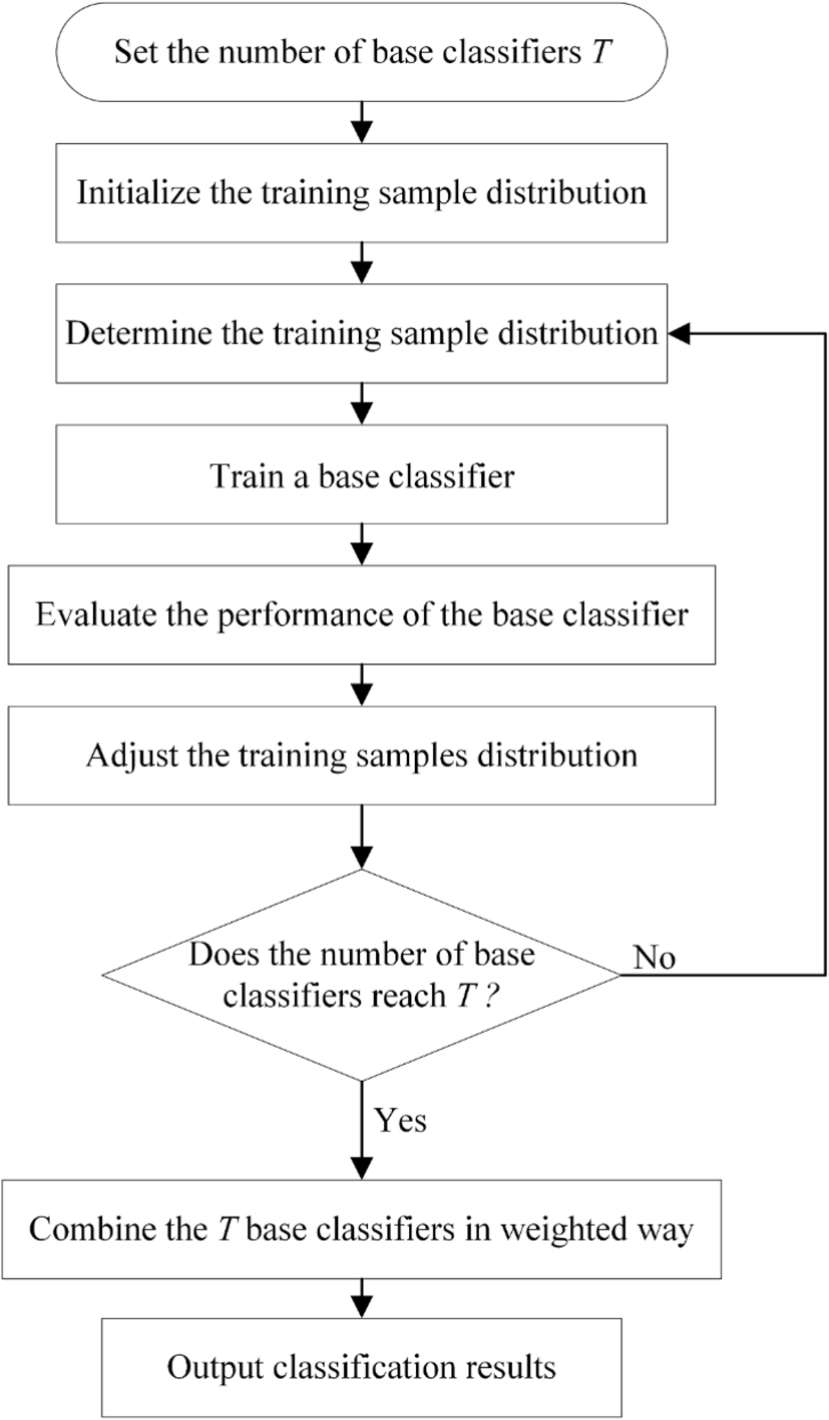
The steps to construct the boosting model.

Boosting model sequentially combines multiple poor learners to build a robust model. The steps to develop the boosting model are presented in Fig. 2. AdaBoost constructs many poor learners from the training data, then linearly synthesizes them into a strong model^41^. Compared with AdaBoost, GBM is a more robust model, which can optimize any differentiable loss function^42^. XGBoost^43^, LightGBM^44^, and CatBoost^45^ are the development and extension of GBM. The detailed comparison between these three models can be referred to previous investigations^46^.

### Combination strategy

This study uses three combination strategies: voting, stacking and bagging to combine multiple models. Figure 4 displays the combination process. Voting is a commonly used method to combine the output of multiple classifiers. In this research, simple soft voting is adopted. Individual classifier $h_{i}$ outputs a $l$ dimensionality vector $(h_{i}^{1} (x),...,h_{i}^{l} (x))^{T}$ when inputting sample $x$. The simple soft voting method calculates and outputs the average value of the output of each classifier.

Bagging uses bootstrap sampling to generate different base classifiers. Given a training dataset with *m* instances, a training subset of *m* instances can be obtained with the replacement sample. Some of the original instances are selected many times, and others are not selected. Repeating the process *t* times, *t* training subsets including *m* instances are obtained. Each training subset is used to develop a base classifier. Voting is adopted to aggregate *t* base classifiers in the classification task.

Stacking combines individual classifiers by training classifier, and the individual classifier is called first-level learner, and the connector is called second-level learner. Stacking first trains the first-level learners utilizing the initial database, then forms a new database to train the second-level learners using the outcomes of the first-level learners as input features and the corresponding initial markers as new markers.

### Bayesian optimization

Bayesian optimization (BO) is suitable for complex problems whose objective function cannot be expressed^47^. BO chooses the next estimation points according to previous outcomes. BO consists of the surrogate model and acquisition function^47^. The goal of the surrogate model is to match the detected points into the objective function. The acquisition function decides to use different points by balancing exploration and exploitation. The Bayesian model can discover the most likely optimum area for the present and avoid missing better parameters in unknown areas.

Gaussian process regression is often chosen as the surrogate model in BO^40^. The acquisition functions include the probability of improvement^48^, expected improvement^49, 50^, and upper/lower confidence bound (UCB/LCB)^51^. To match the acquisition function to the surrogate model, GP-Hedge is introduced to select an appropriate acquisition function in each BO iteration^51, 52^.

### Data

**Figure 3:**
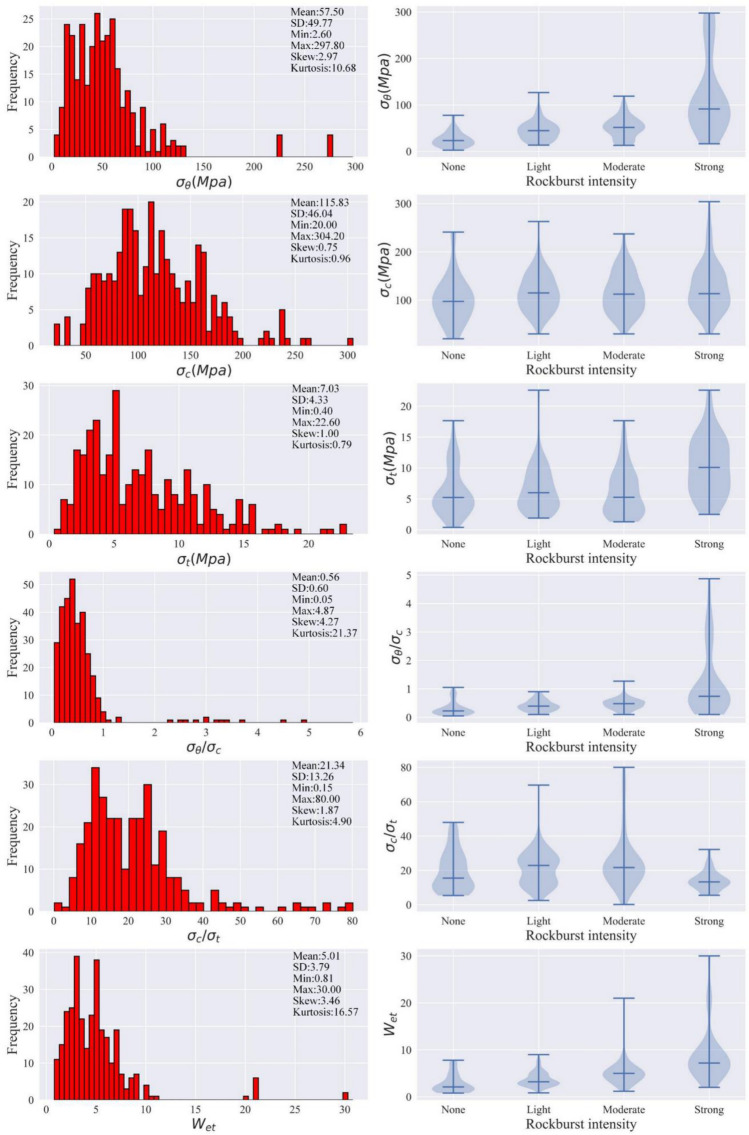
The histograms and violin plots of six variables.

A database including 314 real rockburst cases is established and used for modeling. Table 2 lists different sources of this database. The maximum tangential stress (${\sigma }_{\theta }$), the uniaxial compressive strength (${\sigma }_{c}$), the tensile strength (${\sigma }_{t}$), the stress ratio (${\sigma }_{\theta }/{\sigma }_{c}$), the brittleness ratio (${\sigma }_{c}/{\sigma }_{t}$), and the elastic strain energy index (${W}_{et}$) are selected as the input variables in this study by referring to the previous research^31, 34, 53^. Pearson correlation coefficients (Eq. 3) between the six variables are calculated. Table 3 shows correlation coefficients between variables. Figure 3 displays the statistics and distribution of each variable.

3 $$r = \frac{{\sum\nolimits_{i = 1}^{n} {(X_{i} - \overline{X})(Y_{i} - \overline{Y})} }}{{\sqrt {\sum\nolimits_{i = 1}^{n} {(X_{i} - \overline{X})^{2} } } \sqrt {\sum\nolimits_{i = 1}^{n} {(Y_{i} - \overline{Y})^{2} } } }}$$

**Table 2:** The rockburst database source.

No	Data size	References
1	None(43), Light(78), Moderate(81), Strong(44)	Zhou et al.^23^
2	None(3), Light(7), Moderate(7), Strong(3)	Xue et al.^54^
3	Light(1), Moderate(11)	Pu et al.^26^
4	None(3), Light(4), Moderate(8), Strong(1)	Liu et al.^55^
5	Light(3), Moderate(3)	Jia et al.^56^
6	None(1), Light(2), Strong(4)	Du et al.^57^
7	Light(1), Moderate(5), Strong(1)	Wu et al.^58^
Sum	None(50), light(96), moderate(115), strong(53)

**Table 3:** The correlation coefficient of each variable.

Variables	$\sigma_{\theta }$	$\sigma_{c}$	$\sigma_{t}$	$\sigma_{\theta } /\sigma_{c}$	$\sigma_{c} /\sigma_{t}$	$W_{et}$
$\sigma_{\theta }$	1.00	0.09	0.34	0.90	− 0.26	0.46
$\sigma_{c}$	0.09	1.00	0.47	− 0.25	0.02	0.24
$\sigma_{t}$	0.34	0.47	1.00	0.14	− 0.63	0.35
$\sigma_{\theta } /\sigma_{c}$	0.90	− 0.25	0.14	1.00	− 0.26	0.32
$\sigma_{c} /\sigma_{t}$	− 0.26	0.02	− 0.63	− 0.26	1.00	− 0.13
$W_{et}$	0.46	0.24	0.35	0.32	− 0.13	1.00

**Figure 4:**
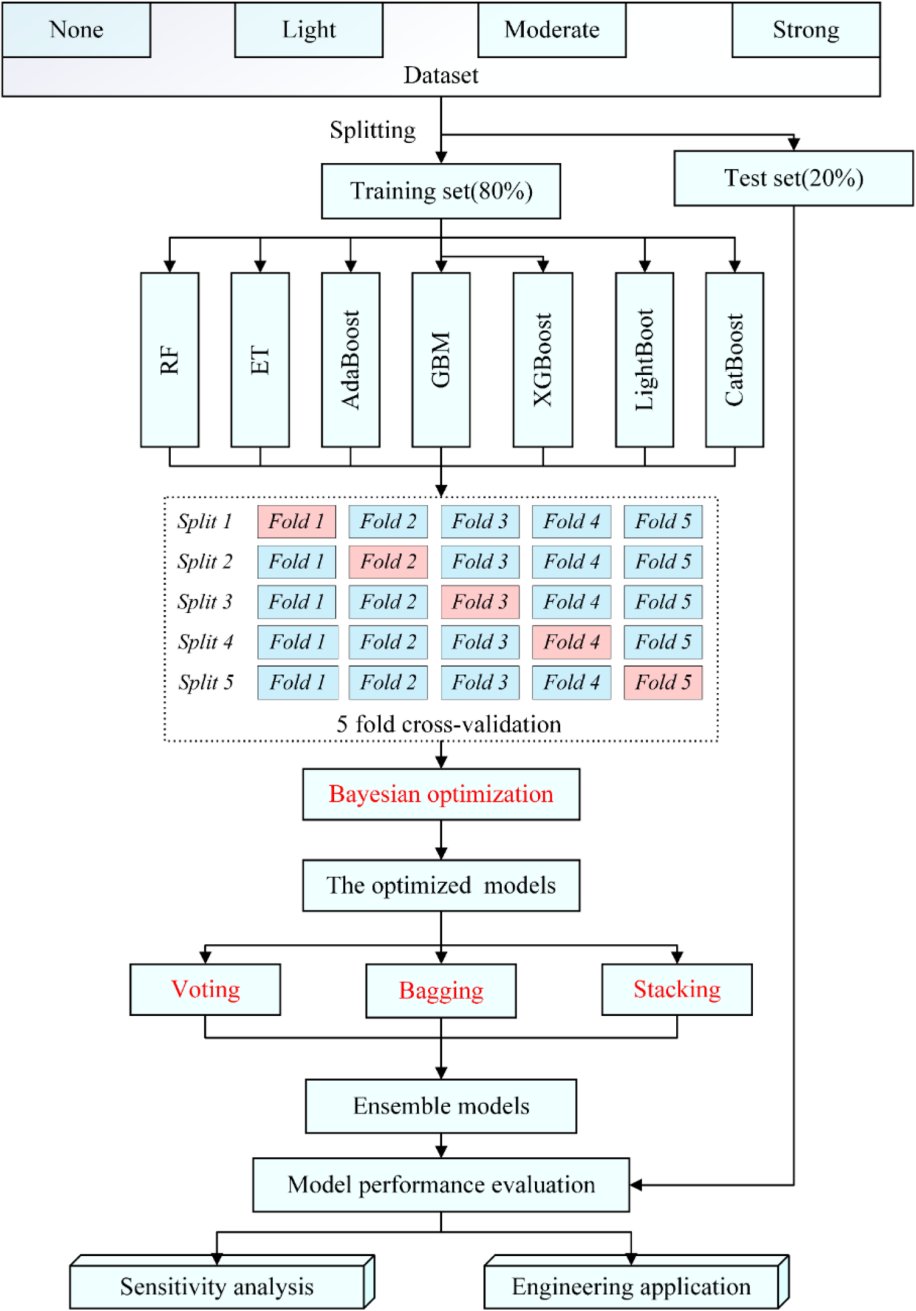
The flowchart of the modeling procedure in this study.

According to Fig. 4, the database is split into a training set (80%) and a test set (20%). The training set is employed to construct seven models based on trees. fivefold cross-validation is employed for model selection. BO is utilized to optimize the hyperparameters of models. The voting, bagging, and stacking strategies are applied to combine these optimized models to develop ensemble models in predicting/evaluating rockburst. The test set is implemented to assess the capability of models. Finally, the optimal model is utilized to conduct the sensitivity analysis and how it can be applied to engineering projects.

## Simulation

### Model metrics

Accuracy was applied to estimate the global performance of the model. The $F_{1}$ combined precision and recall and was utilized to assess the performance of each classification.

4 $$ACC = \frac{1}{m}\sum\nolimits_{i = 1}^{m} {I(\overline{{y_{i} }} = y_{i} )}$$

5 $$Precision = \frac{TP}{{TP + FP}}$$

6 $${\text{Re}} call = \frac{TP}{{TP + FN}}$$

7 $$F_{1} = \frac{2 \times precision \times recall}{{precision + recall}}$$

In Eq. 4, $m$ is the number of samples, $\overline{{y_{i} }}$ represents the predicted labels, $y_{i}$ represents the actual labels, and $I( \cdot )$ is one if the conditions in brackets are true and zero, otherwise. In Eqs. 5 and 6, *TP* is the true positive, *FP* is the false positive, and *FN* is the false negative.

### Model hyperparameters optimization

#### The hyperparameters range

The training set was adopted to train the seven ensemble models based on DTs. Z-score was used to process the input variables (Eq. 8). The open-source *Python* library, *Scikit-learn*^59^, was used to construct RF, ET, AdaBoost, and GBM models. The open-source *Python* libraries, *XGBoost*^43^, *LightGBM*^44^, and *CatBoost*^45^, were utilized to build the XGBoost, LightGBM, and CatBoost models, respectively. Table 4 presents the hyperparameters optimization range in seven models.

8 $$x^{^{\prime}} = \frac{{x - \overline{x}}}{\sigma }$$

**Table 4:** The hyperparameters range.

Model	Hyperparameters	Range of value	References
RF	The number of DTs	(10,100)	Liang et al.^60^
	The DTs maximum depth	(1,10)	
ET	The number of DTs	(10,100)	Liang et al.^60^ and Pedregosa et al.^59^
	The DTs maximum depth	(1,10)	
AdaBoost	The maximum number of DTs	(10,100)	Liang et al.^60^
	Learning rate	(0.01,0.2)	
GBM	The number of boosting iterations	(10,100)	Liang et al.^60^ and Pedregosa et al.^59^
	Learning rate	(0.01,0.2)	
	Maximum depth	(1,10)	
XGBoost	Number of boosting rounds	(10,100)	Liang et al.^60^
	Learning rate	(0.01,0.2)	
LightGBM	Number of boosted DTs	(10,100)	Liang et al.^60^
	Learning rate	(0.01,0.2)	
CatBoost	Max count of DTs	(100,500)	Dorogush et al.^45^
	Learning rate	(0.01,0.2)	

In Eq. 8, $\overline{x}$ is the mean value of the data and $\sigma$ is the standard deviation of the data.

#### The objective function

**Figure 5:**
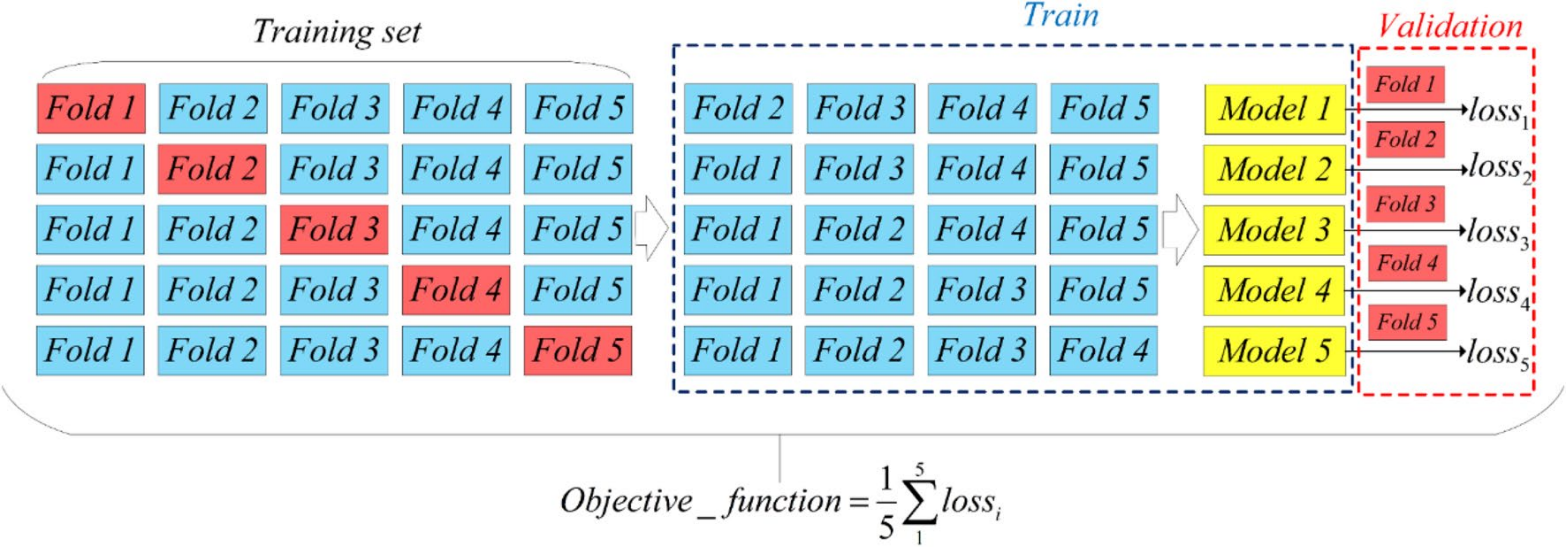
The calculation method of the objective function.

Before hyperparameters optimization, the objective function should be defined. In ML, the cross-entropy loss function is a method to measure classifier performance (Eq. 9). It is generally believed that the classifier performs better when the cross-entropy loss function obtains a smaller value. In this paper, we adopted the cross-entropy loss function in fivefold cross-validation as the objective function. Figure 5 shows the steps to calculate the objective function.

9 $$loss = - \frac{1}{m}\sum\nolimits_{i = 1}^{m} {\log p_{\bmod el} [y_{i} \in C_{{y_{i} }} ]}$$

In Eq. 9**,** $m$ is the number of instances and $p_{\bmod el} [y_{i} \in C_{{y_{i} }} ]$ is the prediction probability of the model in the actual label.

#### The process of BO

**Figure 6:**
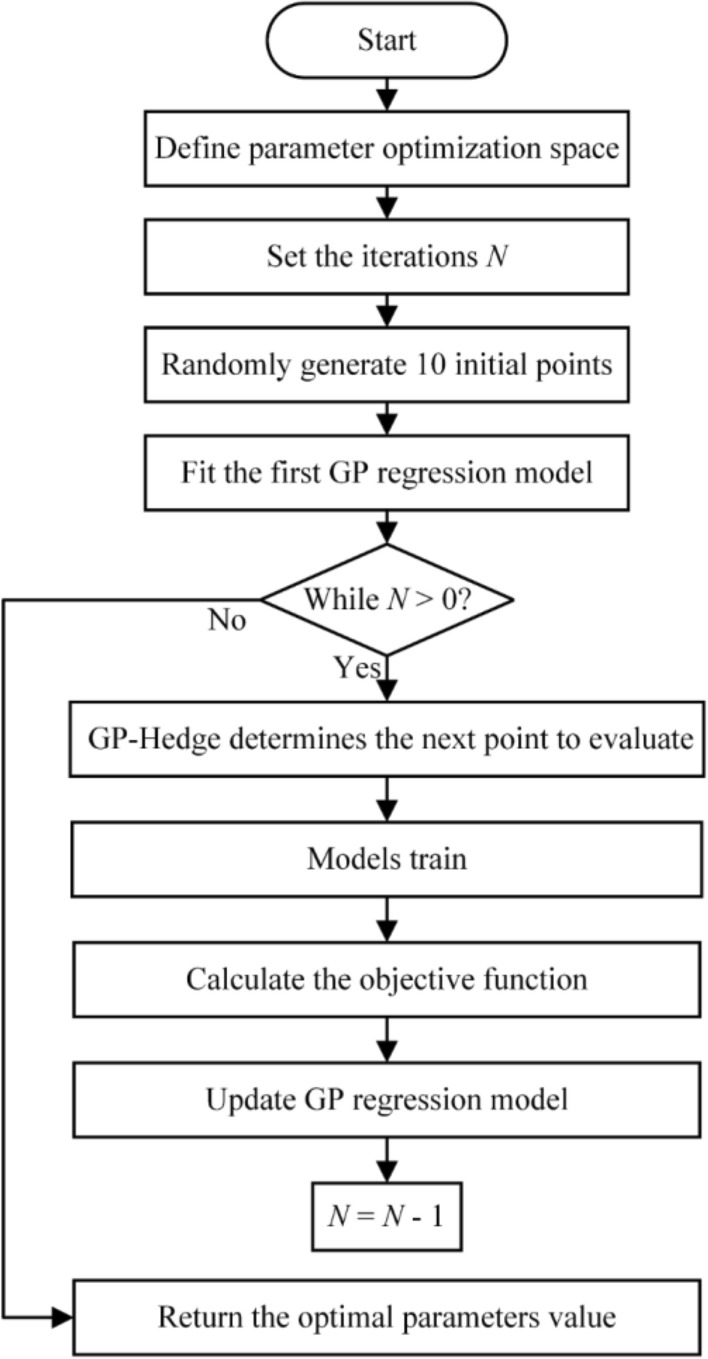
The Bayesian optimization flow chart.

**Figure 7:**
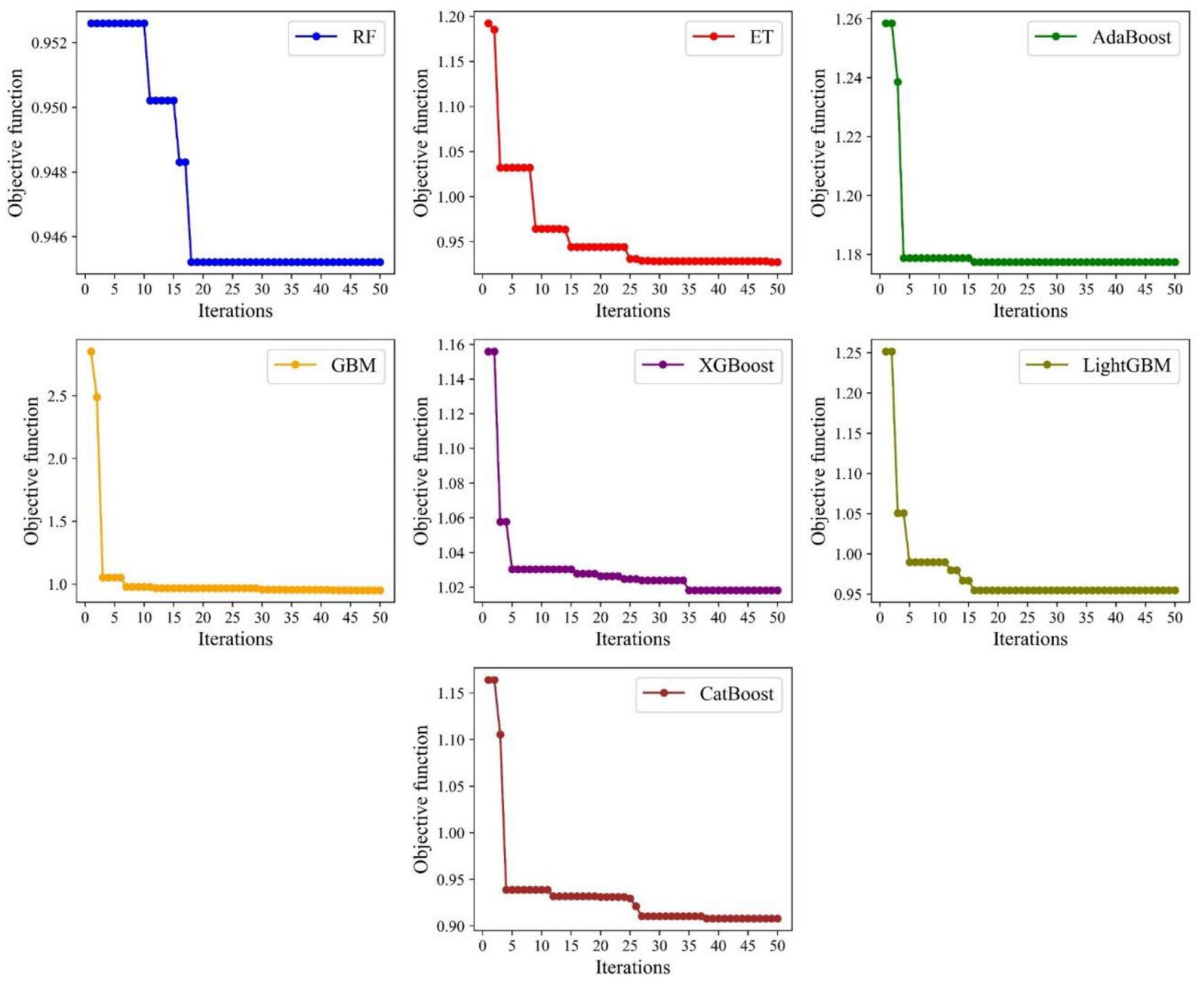
The iterative convergence of the objective function.

In this research, the *Scikit-Optimize* ^61^ was used to perform the BO. The surrogate model in BO adopted the Gaussian process (GP) regression, the acquisition function utilized the GP-Hedge, and the noise was assumed to be Gaussian distribution. The kernel function was an important part of the GP regression. Table 5 tabulates the kernel function parameters of GP regression. Figure 6 illustrates the process that BO optimized the hyperparameters. In this research, the iteration *N* was set to 50. BO can minimize the objective function within the parameter range so that the performance of the model can reach optimum. In addition, Fig. 7 presents the objective function convergence of seven models in 50 iterations. It reflects the variation of the objective function with the iteration process. Different models had different values of the objective function in the initial state, which was related to the random selection of the initial point in BO. With the iteration progress, BO was constantly balancing the process of exploration and utilization, and the value of the objective function was shrinking. After 50 iterations, BO can find the minimum value of the objective function and return the optimum value of hyperparameters. Table 6 shows the optimized parameter value and the training accuracy in seven models. The training accuracies in the seven models varied greatly. XGBoost had the highest training accuracy, and AdaBoost had the worst training performance.

**Table 5:** The kernel function parameters of GP regression in BO.

Kernel	Value	
Matern kernel	The length scale of the kernel	[1, 1, 1]
	Nu	2.5
WhiteKernel	Noise level	1

**Table 6:** The optimized parameter value.

Model	Hyperparameters	Value	Training accuracy
RF	The number of DTs	100	93.23%
	The DTs maximum depth	7	
ET	The number of DTs	88	98.80%
	The DTs maximum depth	10	
AdaBoost	The maximum number of DTs	10	51.39%
	Learning rate	0.195	
GBM	The number of boosting iterations	61	87.25%
	Learning rate	0.0795	
	Maximum depth	2	
XGBoost	Number of boosting rounds	24	99.20%
	Learning rate	0.1258	
LightGBM	Number of boosted DTs	10	85.25%
	Learning rate	0.2	
CatBoost	Max count of DTs	100	92.82%
	Learning rate	0.111	

Nu controlling the smoothness of the learned function.

### Model combination

#### Voting combination

**Figure 8:**
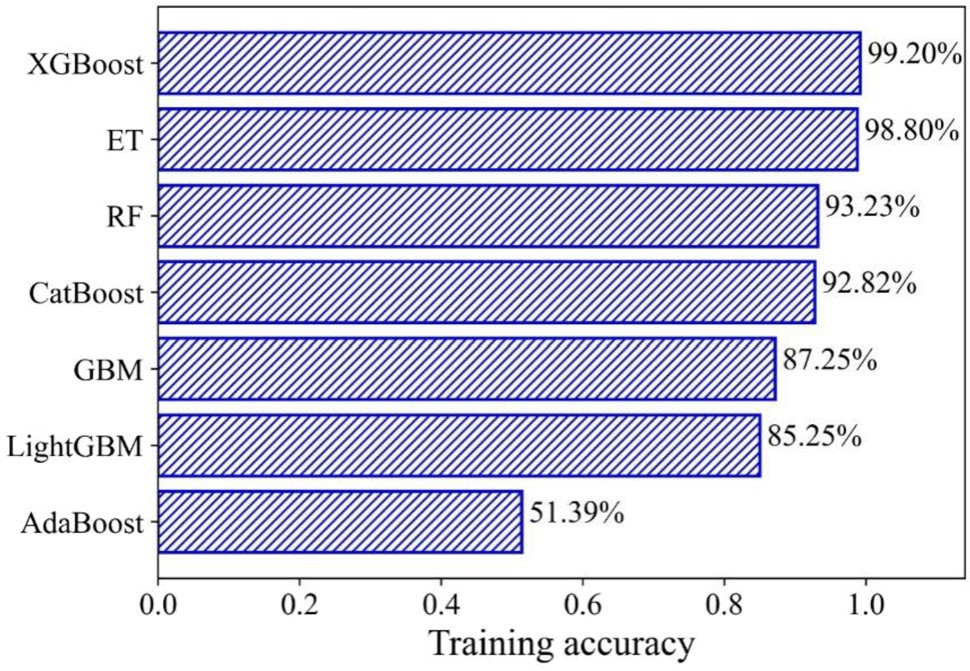
The training accuracy variation of seven models.

According to the accuracy results in the training set (Fig. 8), multiple models were combined by the simple soft voting method. From XGBoost to AdaBoost, the model was added to the voting combination model in order of accuracy in the training set. Table 7 presents the final six voting combination models. It can be seen that with the addition of some models with lower training accuracy, the training accuracy of the voting combination model was gradually decreasing.

**Table 7:** The voting ensemble model.

Model	Base classifier	Training accuracy
Voting 1	XGBoost and ET	99.60%
Voting 2	XGBoost, ET, and RF	98.80%
Voting 3	XGBoost, ET, RF and CatBoost	97.21%
Voting 4	XGBoost, ET, RF, CatBoost and GBM	98.62%
Voting 5	XGBoost, ET, RF, CatBoost, GBM and LightGBM	95.22%
Voting 6	XGBoost, ET, RF, CatBoost, GBM, LightGBM and AdaBoost	95.22%

#### Bagging combination

The seven models were used as base classifiers in bagging ensemble models. Bagging fitted each base classifier on a random subset of the initial training set and then combined their prediction outcomes by voting to build an eventual ensemble model. The number of base estimators in the bagging ensemble model was set to 10. Table 8 displays the final seven bagging combination models. Except for the AdaBoost model, the training accuracies of other models that adopted the bagging combination were reduced.

**Table 8:** The seven bagging ensemble models.

Model	Base classifier	The number of base classifier	Training accuracy
Bagging 1	XGBooost	10	94.82%
Bagging 2	ET	10	96.01%
Bagging 3	RF	10	92.03%
Bagging 4	CatBoost	10	90.83%
Bagging 5	GBM	10	87.25%
Bagging 6	LightGBM	10	83.67%
Bagging 7	AdaBoost	10	53.38%

#### Stacking combination

In stacking combination, we adopted the seven models as the first-level learners, and the second-level learners adopted LR. Like “Voting combination” section, voting combinations, multiple models were combined in turn among first-level learners based on the performance in the training set. Table 9 displays the final seven stacking combination models.

**Table 9:** The stacking ensemble models.

Model	First-level learners	Second-level learner	Training accuracy
Stacking 1	XGBoost	LR	98.00%
Stacking 2	XGBoost and ET	LR	98.40%
Stacking 3	XGBoost, ET, and RF	LR	96.81%
Stacking 4	XGBoost, ET, RF and CatBoost	LR	94.02%
Stacking 5	XGBoost, ET, RF, CatBoost and GBM	LR	94.42%
Stacking 6	XGBoost, ET, RF, CatBoost, GBM and LightGBM	LR	94.02%
Stacking 7	XGBoost, ET, RF, CatBoost, GBM, LightGBM and AdaBoost	LR	94.02%

## Results and discussion

### Model performance evaluation

#### The individual model performance evaluation and comparison

The test set is applied for evaluating the seven base models. Table 10 presents the $F_{1}$ and accuracy of the test set in seven base models. In the individual model, ET and XGBoost perform best, and AdaBoost performs worst. When considering the accuracy in the test set, it can be concluded that the capacity ranking is ET, XGBoost > RF > GBM > CatBoost > LightGBM > AdaBoost. Besides, apart from AdaBoost, these single models have high $F_{1}$ in strong rockburst, and these suggest that ensemble trees have superior capability to forecast massive rockburst hazards.

**Table 10:** The $F_{1}$ and accuracy in seven base models.

Model	Metrics	None	Light	Moderate	Strong
RF	$F_{1}$	0.75	0.82	0.88	0.86
	ACC	84.12%			
ET	$F_{1}$	0.75	0.85	0.90	0.86
	ACC	85.71%			
AdaBoost	$F_{1}$	0.31	0.48	0.50	0
	ACC	42.85%			
GBM	$F_{1}$	0.78	0.72	0.83	0.95
	ACC	80.95%			
XGBoost	$F_{1}$	0.78	0.83	0.89	0.91
	ACC	85.71%			
LightGBM	$F_{1}$	0.75	0.70	0.75	0.90
	ACC	76.20%			
CatBoost	$F_{1}$	0.82	0.75	0.79	0.86
	ACC	79.37%			

**Figure 9:**
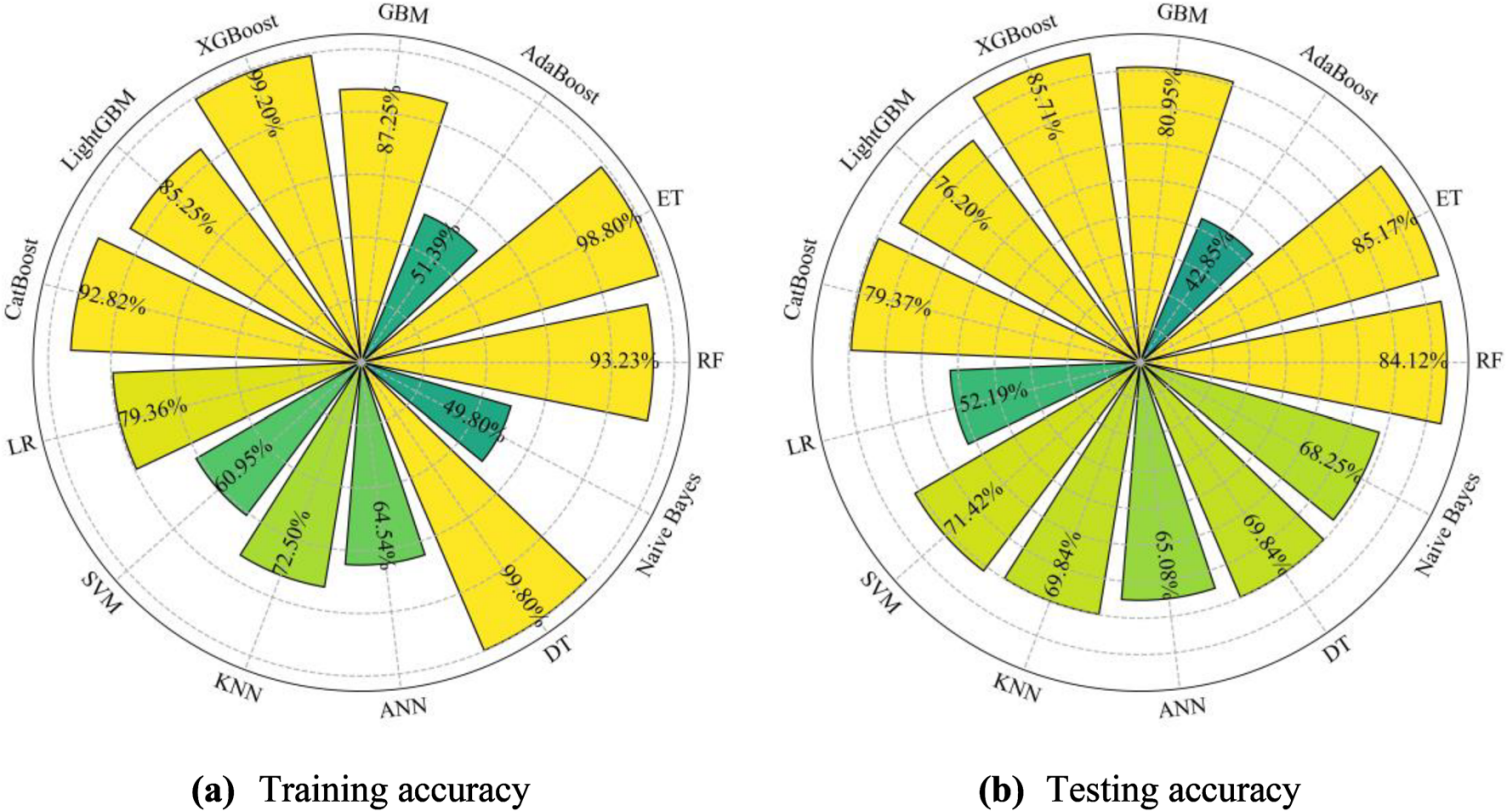
The performance comparison of ensemble trees and other ML models.

Six other widely used ML models, LR, SVM, KNN, ANN, DT, and Naive Bayes, are also developed based on the training set and evaluated by the test set. Their hyperparameters adopt the default value in *Scikit-learn*. Figure 9 shows the performance comparison of ensemble trees and other ML models. DT model suffers from serious overfitting, and the ensemble trees have better generalization than DT. Except for AdaBoost, the ensemble trees have higher testing accuracy than other ML models. These indicate that the proposed ensemble trees solution can get more accurate rockburst prediction results.

#### Investigate the strength of voting combination models

**Figure 10:**
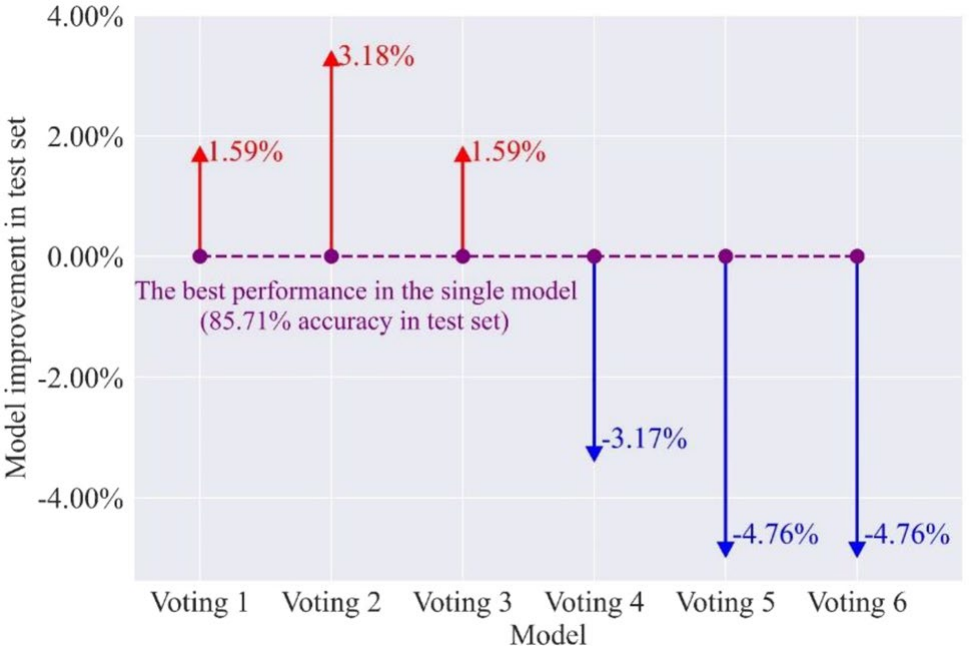
The accuracy improvement of voting combination models in the test set.

**Figure 11:**
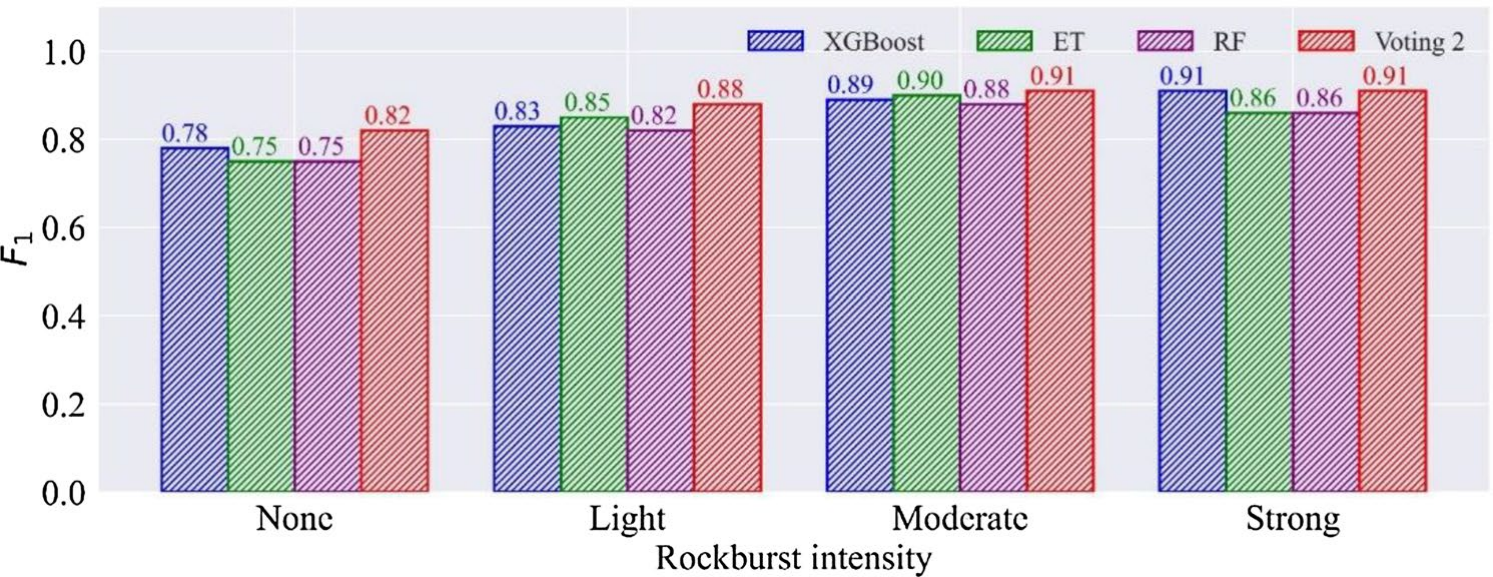
The comparison of $F_{1}$ in voting 2 model and its base classifiers.

Due to the difficulty of selecting base learners, not all voting combination models improve performance compared to their base classifiers. Therefore, the test set is employed to assess the capability of the voting combination models. Table 11 shows the $F_{1}$ and accuracy of the test set in six voting combination models. It is found that the voting 2 has an outstanding $F_{1}$ in a single type of rockburst prediction and the highest accuracy. Besides, six voting combination models have the same performance in predicting strong rockburst. Figure 10 presents the accuracy improvement of the voting combination model in the test set compared to the individual optimal classifier. Voting 1, voting 2, and voting 3 perform better than the individual model on the testing set. The performances of the three models increase by 1.59%, 3.18%, and 1.59%, respectively. With the combination of poor performance base learners, voting 4, voting 5, and voting 6 perform poorly than the single optimal model. Figure 11 compares the $F_{1}$ of the voting 2 model and its base classifiers. It can be seen that voting 2 has better performance than XGBoost, ET, and RF models in the single rockburst category. The results suggest that the voting 2 combining high accuracy and diversity is the best in voting combination models.

**Table 11:** The $F_{1}$ and accuracy in voting combination models.

Model	Metrics	None	Light	Moderate	Strong
Voting 1	$F_{1}$	0.82	0.86	0.89	0.91
	ACC	87.30%			
Voting 2	$F_{1}$	0.82	0.88	0.91	0.91
	ACC	88.89%			
Voting 3	$F_{1}$	0.82	0.86	0.89	0.91
	ACC	87.30%			
Voting 4	$F_{1}$	0.75	0.78	0.85	0.91
	ACC	82.54%			
Voting 5	$F_{1}$	0.75	0.75	0.83	0.91
	ACC	80.95%			
Voting 6	$F_{1}$	0.75	0.75	0.83	0.91
	ACC	80.95%

#### The effect of bagging integration on single model performance

**Figure 12:**
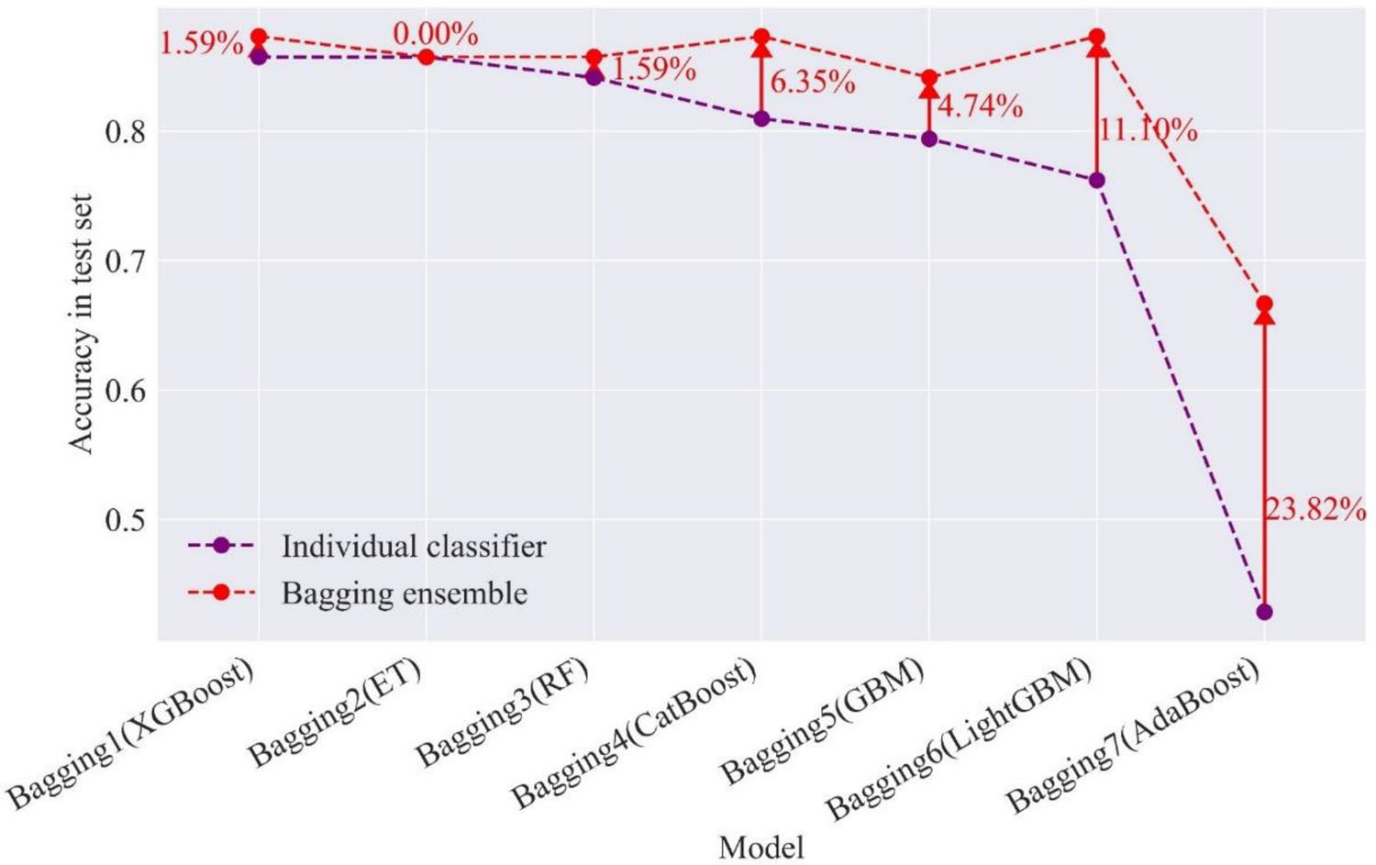
The accuracy improvement of bagging combination models in the test set.

**Figure 13:**
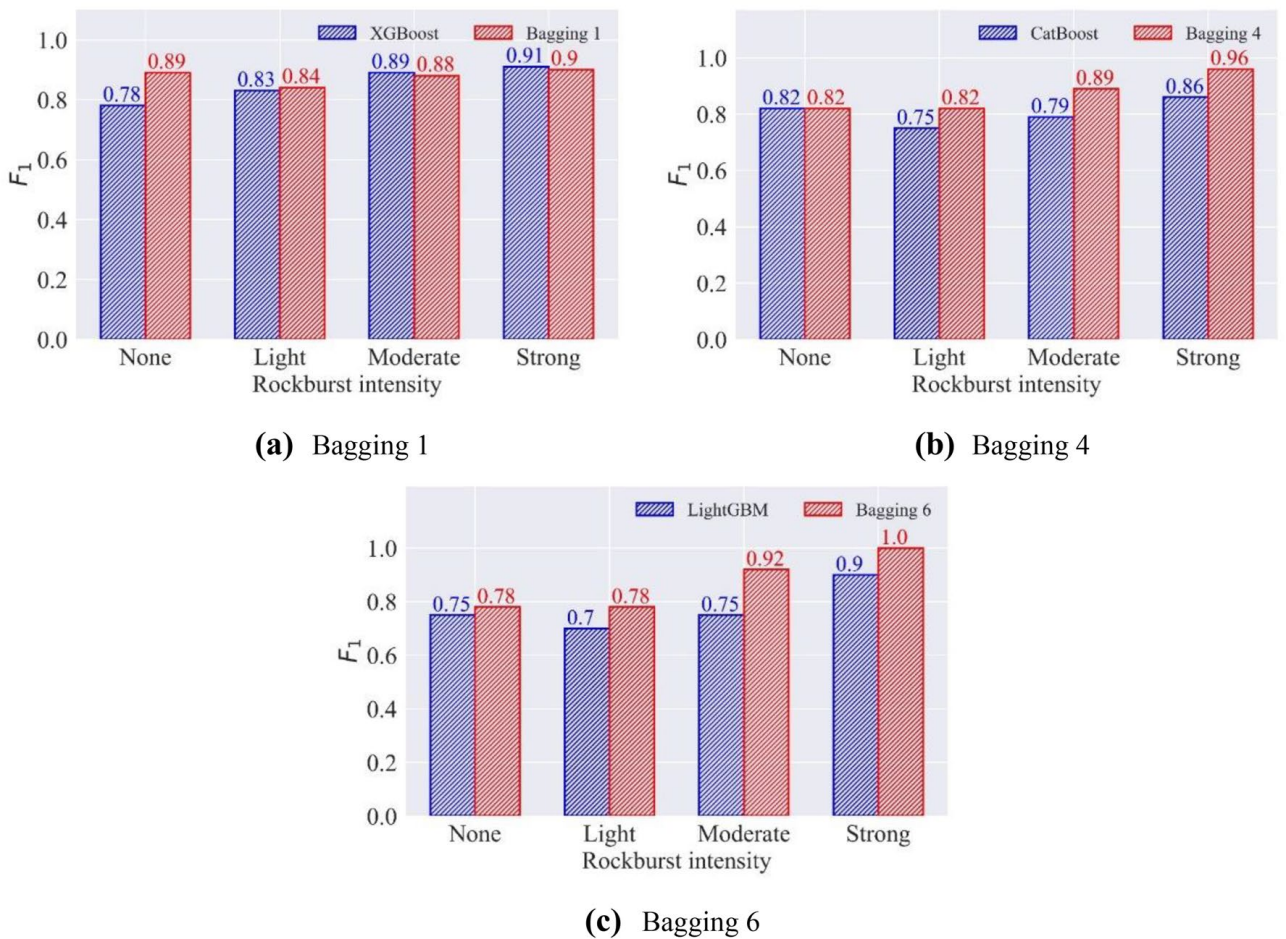
The comparison of $F_{1}$ in bagging 1, bagging 4, and bagging 6 and their base classifiers.

Bagging combines independent base classifiers, which reduces the error. The bagging combination models are evaluated by the test set to determine the enhancement of models (i.e., RF, ET, etc.) performance before and after bagging integration. Table 12 presents the $F_{1}$ and accuracy of the test set in the seven bagging combination models. Figure 12 displays the accuracy improvement of the bagging combination model in the test set compared to the individual classifier. After the bagging combination, except for the ET model, the accuracies in the test set of other models increase, and the accuracy of the AdaBoost increases by 23.82%. XGBoost, GBM, and LightGBM achieve the best performance after adopting the bagging combination. Figure 13 compares the $F_{1}$ of XGBoost, GBM, and LightGBM and their bagging combination models. The bagging 1 that adopts XGBoost as the base learner has great improvement for predicting the none intensity of rockburst. Bagging 4 and bagging 6 perform better than their base classifiers in the prediction of a single rockburst category.

**Table 12:** The $F_{1}$ and accuracy in seven bagging combination models.

Model	Metrics	None	Light	Moderate	Strong
Bagging 1	$F_{1}$	0.89	0.84	0.88	0.90
	ACC	87.30%			
Bagging 2	$F_{1}$	0.82	0.84	0.86	0.90
	ACC	85.71%			
Bagging 3	$F_{1}$	0.75	0.79	0.90	0.95
	ACC	85.71%			
Bagging 4	$F_{1}$	0.82	0.82	0.89	0.96
	ACC	87.30%			
Bagging 5	$F_{1}$	0.78	0.77	0.87	0.95
	ACC	84.13%			
Bagging 6	$F_{1}$	0.78	0.78	0.92	1.00
	ACC	87.30%			
Bagging 7	$F_{1}$	0.75	0.50	0.71	0.71
	ACC	66.67%

#### Explore the power of the stacking combination models

**Figure 14:**
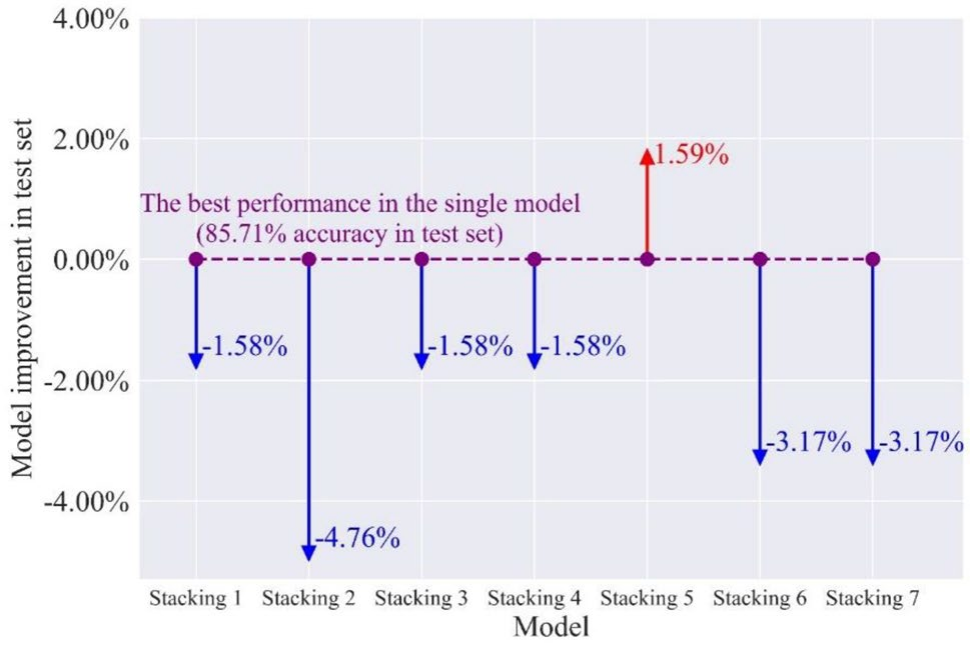
The accuracy improvement of stacking combination models in the test set.

**Figure 15:**
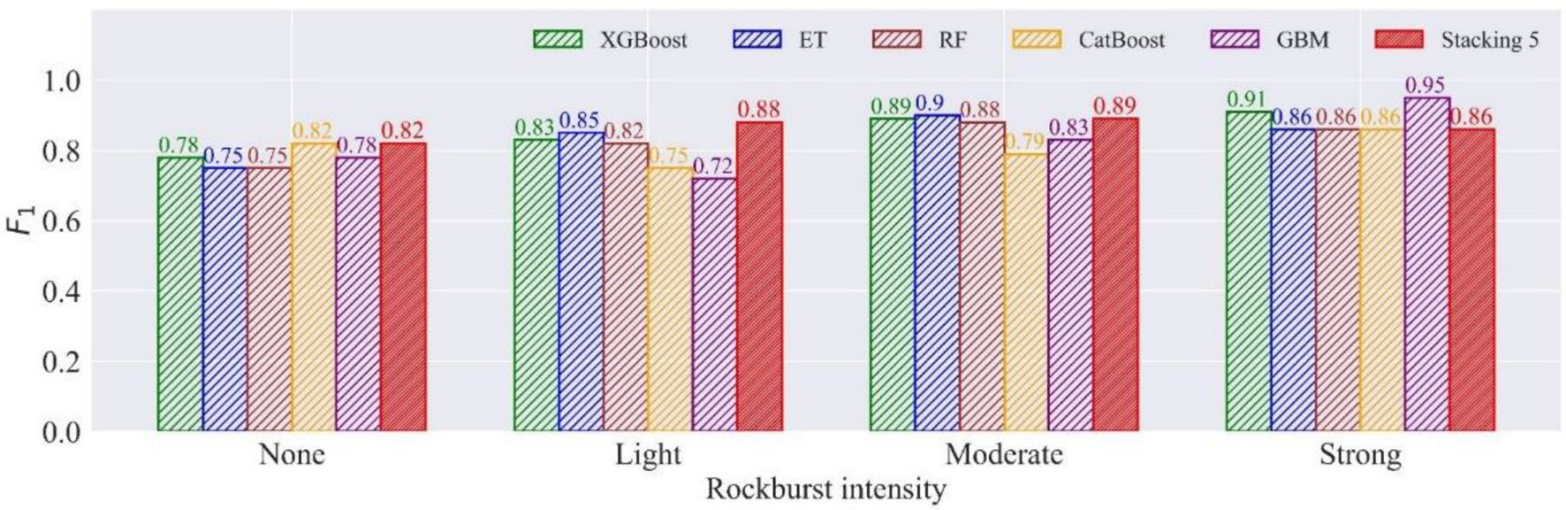
The comparison of $F_{1}$ in stacking 5 and its base classifiers.

Stacking is a learning combination method, and it is of importance to match the appropriate first-level learners (i.e., RF, ET, etc.) to second-level learner (i.e., LR). The performance of stacking combination models in the test set can reflect whether the model combination is appropriate. Table 13 shows the $F_{1}$ and accuracy of the test set in seven stacking combination models. Stacking 5 is the optimal model in terms of accuracy and $F_{1}$. Figure 14 illustrates the accuracy improvement of the stacking combination models in the test set. Compared with the previous two combination strategies, the rockburst prediction performance of the stacking combination with the individual classifier is not ideal, and only the stacking 5 performs better than the individual optimal classifier in the seven stacking combination models. Figure 15 compares the $F_{1}$ of stacking 5 and its base classifiers. Stacking 5 has an improvement in predicting the rockburst of light intensity compared to its base classifiers. Contrasted to GBM, the performance of stacking 5 in the strong rockburst prediction is weakened. We assume that the reason for the poor performance of the stacking models might be that the LR does not validly match models based on DTs.

**Table 13:** The $F_{1}$ and accuracy in stacking combination models.

Model	Metrics	None	Light	Moderate	Strong
Stacking 1	$F_{1}$	0.82	0.86	0.85	0.80
	ACC	84.13%			
Stacking 2	$F_{1}$	0.75	0.83	0.84	0.74
	ACC	80.95%			
Stacking 3	$F_{1}$	0.75	0.83	0.87	0.86
	ACC	84.13%			
Stacking 4	$F_{1}$	0.75	0.83	0.87	0.86
	ACC	84.13%			
Stacking 5	$F_{1}$	0.82	0.88	0.89	0.86
	ACC	87.30%			
Stacking 6	$F_{1}$	0.82	0.83	0.83	0.80
	ACC	82.54%			
Stacking 7	$F_{1}$	0.82	0.83	0.83	0.80
	ACC	82.54%

## Summary

For this part, seven ensemble trees are evaluated and compared with other ML models. Except for AdaBoost, the proposed ensemble trees have superior rockburst estimation results than other ML models. The XGBoost and ET perform best in the single models, and the accuracies in the test set are 85.7%. In the voting combination models, the voting 2 consisting of XGBoost, ET, and RF, is the best, and the accuracy in the test set is obtained 88.89%. The bagging combination models which adopt XGBoost, CatBoost, and LightGBM as base classifiers are optimal and their accuracies in the test set are obtained as 87.30%. In stacking combination models, the stacking 5, which utilizes XGBoost, ET, RF, CatBoost, and GBM as first-level learners and LR as the second-level learner, has the most outstanding performance, and the accuracy in the test set is achieved as 87.30%. It is found that voting 2 is the best model for rockburst prediction in all proposed models.

### Analysis of the adaptation and superiority of applying combination model

**Figure 16:**
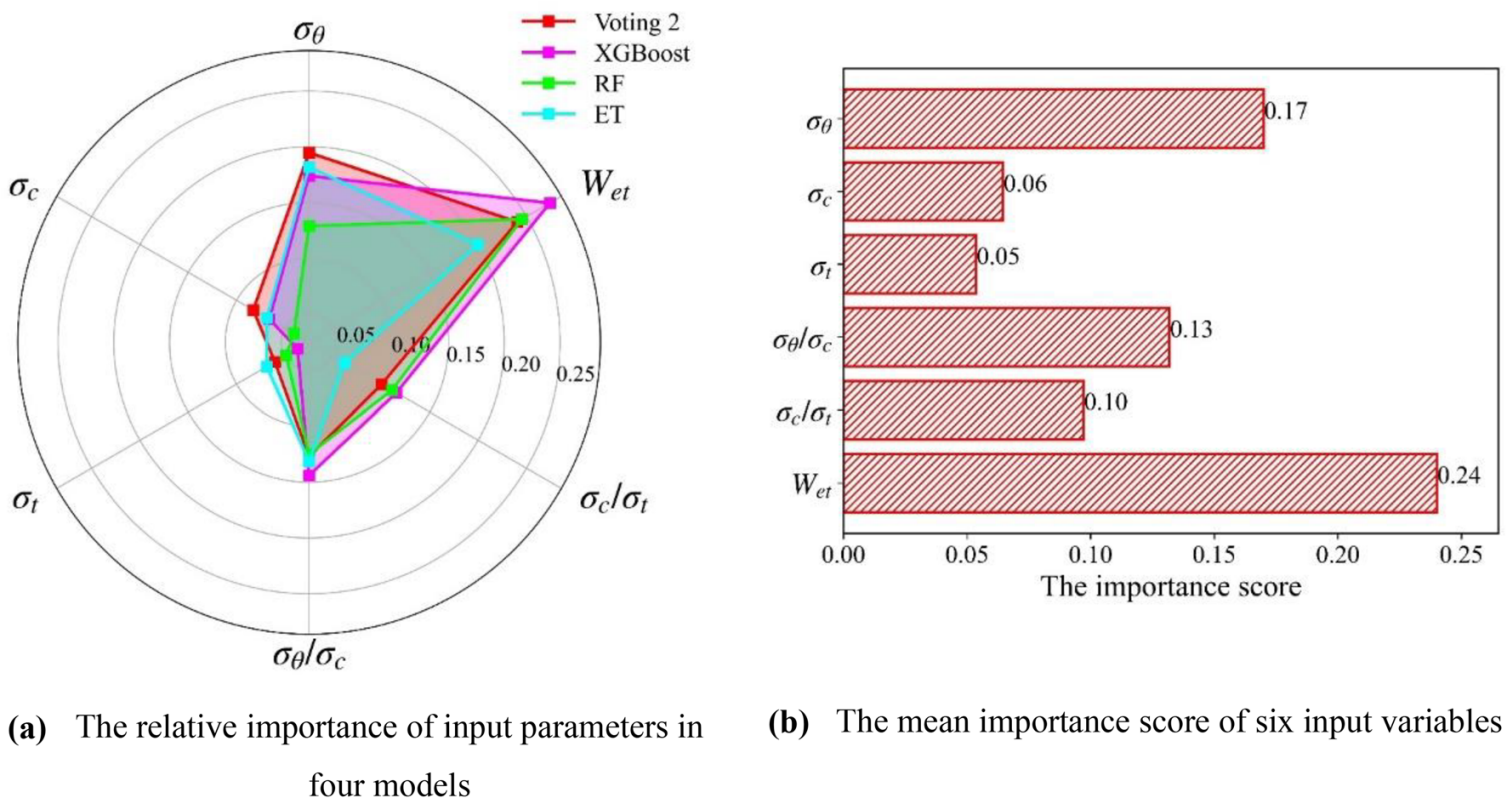
The relative importance of input parameters.

In the previous section, we find that voting 2 is the most excellent combination model, and XGBoost, ET, and RF are the best three single models. In this part, sensitivity analysis is conducted to determine the adaptation and superiority of applying the voting 2 model for rockburst cases. The permutation feature importance algorithms^59^ are introduced to discover the crucial input parameters affecting rockburst. The relative importance of input parameters in voting 2 and its base classifiers are calculated, as shown in Fig. 16a. Although the variables with less importance are different in the four models, the pivotal variables are consistent. The relative importance scores of input variables in the four models are averaged. Figure 16b displays the mean importance score of the six input variables. The importance ranking of parameters influencing the rockburst is $W_{et}$ > $\sigma_{\theta }$ > $\sigma_{\theta } /\sigma_{c}$ > $\sigma_{c} /\sigma_{t}$ > $\sigma_{c}$ > $\sigma_{t}$. The $W_{et}$ is the most critical factor that affected the rockburst. Energy-absorbing bolts and pressure relief blasting can be implemented to absorb the strain energy in deep excavation engineering to prevent rockburst^62^.

**Figure 17:**
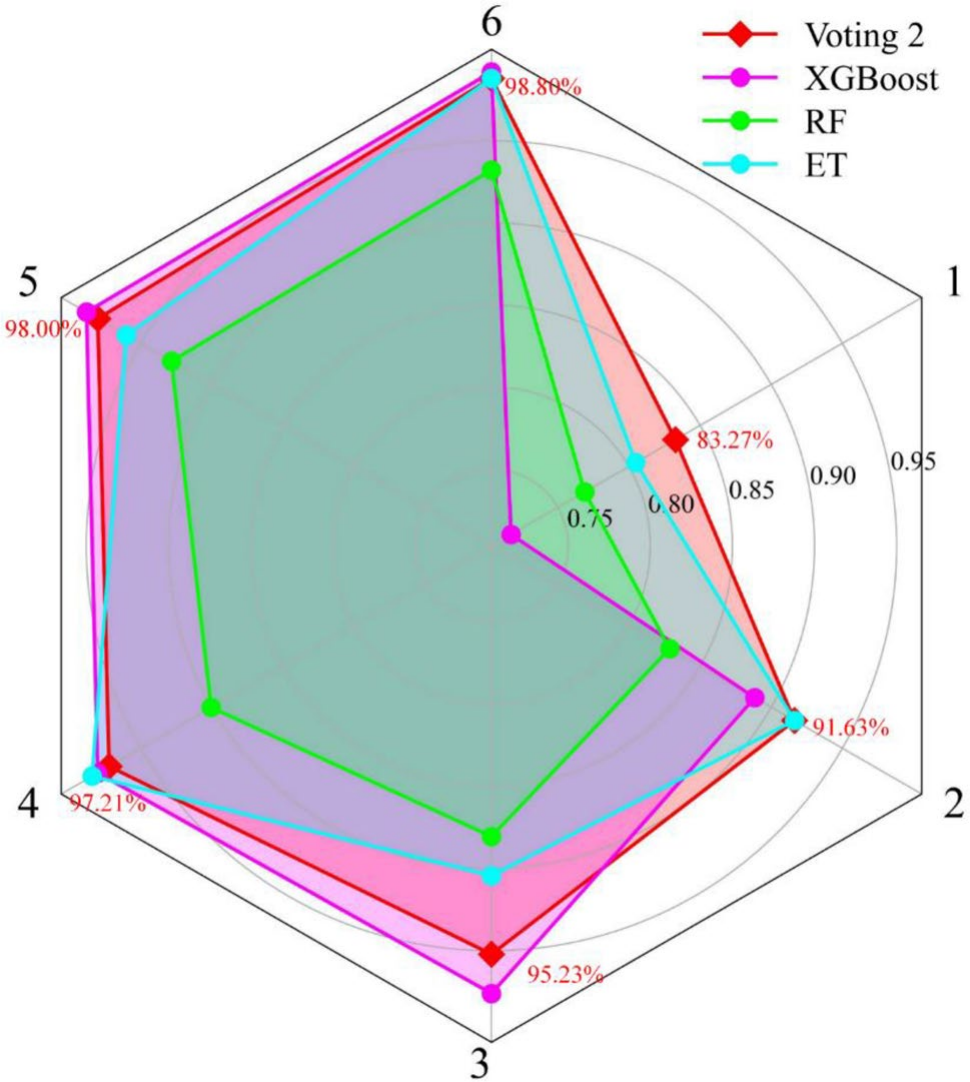
The accuracy in training set when adopting different input parameters.

**Figure 18:**
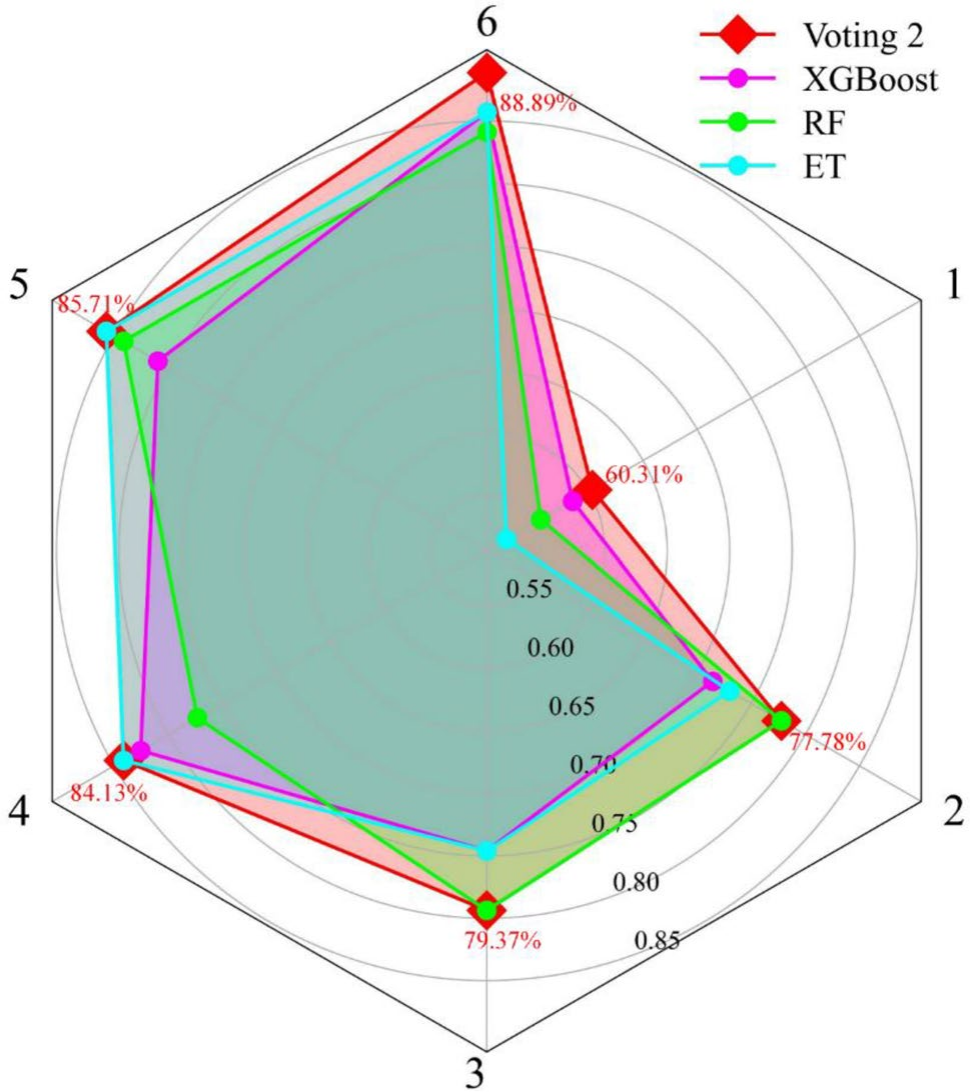
The accuracy in the test set when adopting different input parameters.

To inspect the adaptation and superiority of voting 2, the number of input parameters are varied, and the performances in voting 2 and three base classifiers are recorded and compared. According to the importance of variables influencing rockburst, some variables are reduced based on the original training and test sets to generate five datasets. Table 14 lists the variations of input parameters and generated five datasets. The five datasets are used to train and evaluate voting 2, XGBoost, RF, and ET. Table 15 tabulates the training and test results of four models in six datasets with different input parameters. According to Fig. 17, with the change of input parameters, the performance of the voting 2 in the training set is close or better to the single optimal model. Depending on Fig. 18, it can be seen that the single models have great differences in capacities for predicting rockburst with the variation of input parameters. For instance, XGBoost performs best for rockburst evaluation in 6 input parameters, but in the absence of ${\sigma }_{c}$, the estimation results of XGBoost can be worse. On the contrary, although RF has optimal performance in only 3 and 2 input parameters, it performs worse than XGBoost with the increase of input parameters. As for ET, when only ${W}_{et}$ is available for evaluating rockburst, it is not a good model. In practical engineering, some input parameters are difficult to obtain or missing, and adopting the single model for rockburst prediction might lead to disappointing outcomes. By contrast, the voting 2 model always has the optimal capability in the test set with different input parameters and can deal with the variation or missing of input data.

**Table 14:** The variation of input parameters.

Datasets	The number of input variables	The input variables
Original dataset	6	$W_{et}$, $\sigma_{\theta }$, $\sigma_{\theta } /\sigma_{c}$, $\sigma_{c} /\sigma_{t}$, $\sigma_{c}$, and $\sigma_{t}$
Dataset 1	5	$W_{et}$, $\sigma_{\theta }$, $\sigma_{\theta } /\sigma_{c}$, $\sigma_{c} /\sigma_{t}$, and $\sigma_{c}$
Dataset 2	4	$W_{et}$, $\sigma_{\theta }$, $\sigma_{\theta } /\sigma_{c}$, and $\sigma_{c} /\sigma_{t}$
Dataset 3	3	$W_{et}$, $\sigma_{\theta }$, and $\sigma_{\theta } /\sigma_{c}$
Dataset 4	2	$W_{et}$ and $\sigma_{\theta }$
Dataset 5	1	$W_{et}$

**Table 15:** The rank system for six datasets with different input parameters.

Model	Type	Original dataset		Dataset 1		Dataset 2		Dataset 3		Dataset 4		Dataset 5		Total Rank	Final Rank
		ACC	Rank	ACC	Rank	ACC	Rank	ACC	Rank	ACC	Rank	ACC	Rank		
Voting 2	Training	98.80%	2	98.01%	3	97.21%	2	95.22%	3	91.63%	4	83.27%	4	18	38
	Testing	88.89%	4	85.71%	3	84.13%	3	79.37%	3	77.78%	3	60.32%	4	20	
XGBoost	Training	99.20%	4	98.80%	4	98.01%	3	97.61%	4	88.84%	2	71.71%	1	18	28
	Testing	85.71%	2	80.95%	1	82.54%	2	74.60%	1	71.43%	1	58.49%	3	10	
RF	Training	93.23%	1	92.83%	1	90.04%	1	88.05%	1	82.87%	1	76.89%	2	7	19
	Testing	84.13%	1	84.13%	2	77.30%	1	79.37%	3	77.78%	3	55.56%	2	12	
ET	Training	98.80%	2	96.02%	2	98.41%	4	90.44%	2	91.03%	3	80.48%	3	16	29
	Testing	85.71%	2	85.71%	3	84.13%	3	74.60%	2	73.02%	2	52.38%	1	13

A ranking system^63^ is introduced to evaluate the performance of four models in different training and test sets comprehensively, as shown in Table 15. The training and testing accuracies of four models in the same dataset are ranked. The higher the accuracy, the higher the ranking score. The total rank in a model is obtained by adding the ranks considering the whole six datasets. The final rank is the sum of total ranks in training and test sets. The voting 2 has the highest final rank, indicating that the combination model has the most remarkable capacity in training and testing phases with different input parameters. The results suggest that the voting 2 has better robustness than single models and can copy with polytropic engineering environments.

### Engineering application

**Figure 19:**
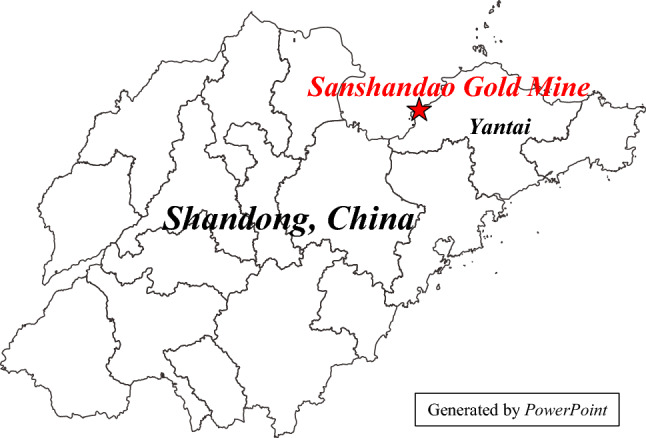
The location of Sanshandao Gold Mine.

**Figure 20:**
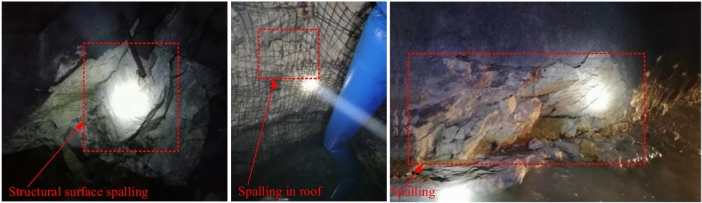
The rockburst site in Sanshandao Gold Mine^62^.

**Figure 21:**
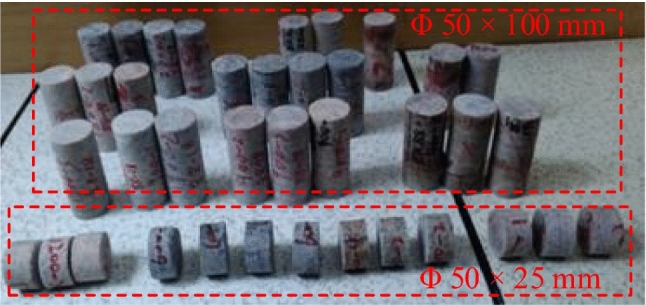
The processed rock samples.

**Figure 22:**
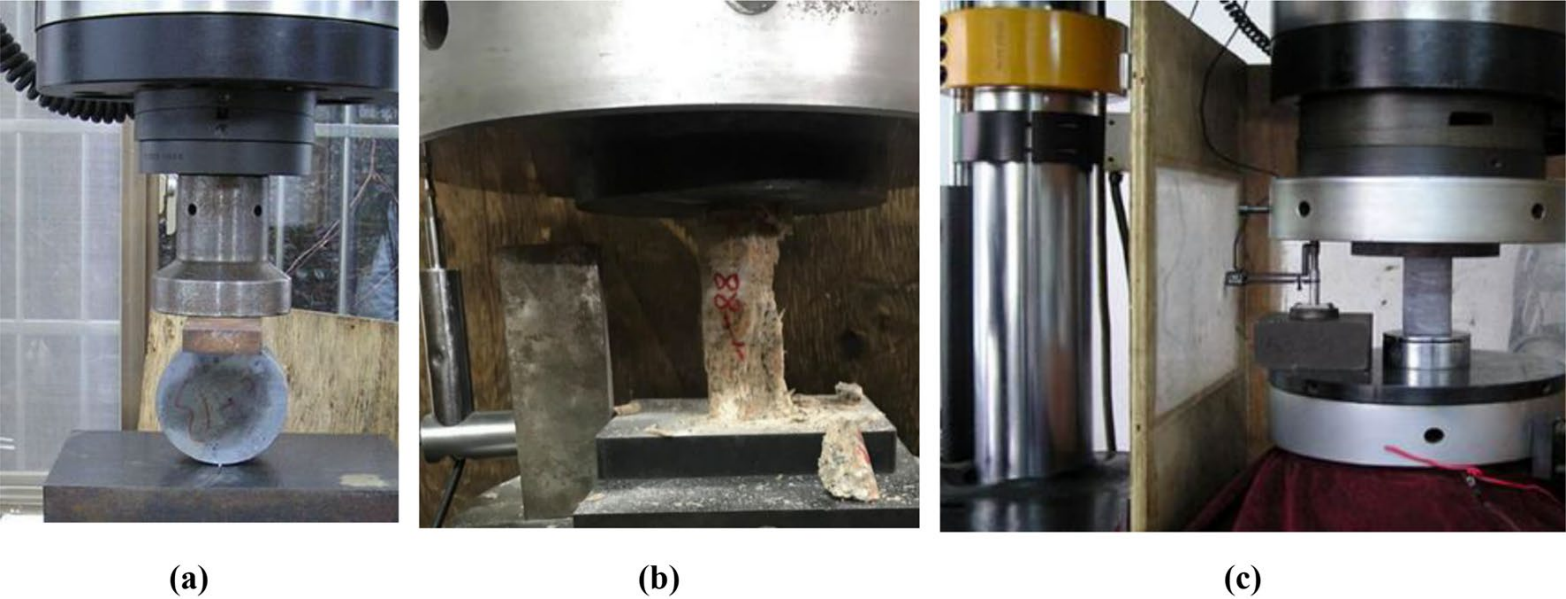
The rock mechanics tests in the laboratory: (**a**) Split tension test, (**b**) Uniaxial compression experiment, (**c**) Rock loading and unloading test.

The Sanshandao Gold Mine is located in Shandong Province, China, as shown in Fig. 19. To meet the production needs, the production of the Sanshandao Gold Mine is going deeper strata. Under the deep and high-stress environment, rockburst is a geological hazard threatening mine production. Figure 20 shows some rockburst sites in Sanshandao Gold Mine. To carry out the rockburst assessment, eight groups of rock specimens from eight locations in the Sanshandao Gold Mine were carried out in rock mechanics experiments. According to the test requirements, the rock samples were processed into two specifications of Φ 50 × 100 mm and Φ 50 × 25 mm, as shown in Fig. 21. The Brazilian splitting tensile tests were carried out by the INSTRON 1342 rock mechanics test system with the rock samples of Φ 50 × 25 mm. The uniaxial compression tests and the loading and unloading tests utilized the INSTRON 1346 rock mechanics test system with the rock samples of Φ 50 × 100 mm. Figure 22 illustrates these three rock mechanics experiments.

$\sigma_{c}$, $\sigma_{t}$, and $W_{et}$ were obtained by rock mechanics experiments, and $\sigma_{\theta }$ was calculated according to the stress of the surrounding rock. Through field observation and evaluation, the rockburst grade was obtained. Table 16 tabulates the rock mechanical parameters and rockburst grade in eight different regions.

**Table 16:** Rock mechanics parameters and rockburst grade in Sanshandao Gold Mine.

No	Rock type	$\sigma_{\theta }$	$\sigma_{c}$	$\sigma_{t}$	$\sigma_{\theta } /\sigma_{c}$	$\sigma_{c} /\sigma_{t}$	$W_{et}$	Grade
1	Gabbro	28.4	59.65	8.67	0.48	6.88	2.12	Light
2	Gabbro	36.58	90.47	14.88	0.40	6.08	2.11	Light
3	Granite	55.52	115.65	12.15	0.48	9.52	1.9	Light
4	Gabbro	71.15	131.477	14.91	0.54	8.82	5.46	Moderate
5	Granite	86.93	165.23	13.44	0.53	12.29	9.1	Moderate
6	Granite	113.56	115.65	9.46	0.98	12.23	3.99	Strong
7	Granite	36.46	110.01	12.94	0.33	8.50	4.53	Moderate
8	Granite	97.06	97.51	11.93	1.00	8.17	4.93	Strong

To verify the practicability of the combination model, the voting 2 is applied to predict rockburst in Sanshandao Gold Mine. Meanwhile, four empirical criteria methods are used for rockburst prediction, as shown in Table 17. Additionally, Table 18 presents the rockburst prediction results. The voting 2 model has the best performance with 100% accuracy compared to the other methods, which suggests the combination model has superior engineering practicability.

**Table 17:** Empirical method index.

Empirical method	Classification criteria				
	Index	None	Light	Moderate	Strong
Russenes criterion^8^	$\sigma_{\theta } /\sigma_{c}$	≤ 0.2	0.2–0.3	0.3–0.55	> 0.55
$\sigma_{c}$^64^	$\sigma_{c}$	< 80	80–120	120–180	> 180
Rock brittleness coefficient^65^	$\sigma_{c} /\sigma_{t}$	> 40	40–26.7	26.7–14.5	< 14.5
Strain energy storage index^66^	$W_{et}$	< 2.0	2.0–3.5	3.5–5.0	> 5.0

**Table 18:** The rockburst prediction results in Sanshandao Gold Mine.

No	Russenes criterion	$\sigma_{c}$	Rock brittleness coefficient	Strain energy storage index	Voting 2	Actual grade
1	M	N	S	L	L	L
2	M	L	S	L	L	L
3	M	L	S	N	L	L
4	M	M	S	S	M	M
5	M	M	S	S	M	M
6	S	L	S	M	S	S
7	M	L	S	M	M	M
8	S	L	S	M	S	S
Accuracy	62.5%	50%	25%	37.5%	100%	

N = none rockburst, L = light rockburst, M = moderate rockburst, S = strong rockburst.

## Conclusion

1. This study comprehensively introduced and evaluated the application of the seven ensemble trees in rockburst prediction. The performance ranking of seven models is XGBoost and ET > RF > GBM > CatBoost > LightGBM > AdaBoost. Except for AdaBoost, in the tree-based models, the testing accuracy ranges (76.2%, 85.71%) and $F_{1}$ of strong rockburst ranges (0.86, 0.91). The ensemble trees have superior capacities than other ML models in general. Besides, the ensemble tree models are beneficial to forecast the occurrence of strong rockburst for protecting the safety of workers and facilities in underground engineering. Not only that, these tree-based models have fewer parameters to tune, and they are easy to apply to the field.
2. To improve robustness and capability, three combination strategies, including voting, bagging, and stacking, were used to combine multiple models. The testing accuracy of voting combination models is range (80.95%, 88.89%), testing accuracy of bagging combination models is range (66.67%, 87.3%), and testing accuracy of stacking combination models is range (80.95%, 87.3%). The combination models have better capacity than single models, and they are suitable for huge and expensive projects that need to forecast rockburst precisely. It is worth noting that the voting 2 model, which adopts simple soft voting to combine XGBoost, RF, and ET, has an accuracy of 88.89% and is the most excellent in all models.
3. Sensitivity analysis is applied to analyze the adaptation and strength of the voting 2 model compared to single models. The single model has different performances with different input parameters and is susceptible to the variation of parameters. In contrast, the combination model (i.e., voting 2) has better robustness and can receive the optimal capability when the input parameters vary. The results suggest that the combination model has better applicability for rockburst evaluation on-site when some parameters miss or are difficult to obtain.
4. The real rockburst cases from Sanshandao Gold Mine, China, are measured and recorded. These datasets validate the practicability (100% accuracy) and advantage of the voting 2 model compared to empirical methods. Furthermore, the validation data can be employed to expand the rockburst database for building more strong models in the future.
5. The limitations of this study are that the performance of the stacking ensemble models could not achieve the desired effect. The second-level learner only considers the LR model in this paper, which is narrow, and it is necessary to explore appropriate second-level learners to match tree models in the future. It consumes more time and computing power to train the combination models than the single models, and fortunately, the limitation can be solved with the development of computation techniques.

## Supplementary Information

Supplementary Information.
