# Supplementary material for: Novel ensemble intelligence methodologies for rockburst assessment in complex and variable environments
Source: Sci Rep. 2022 Feb 3;12:1844. doi: 10.1038/s41598-022-05594-0 (PMC8814189; doi:10.1038/s41598-022-05594-0)
Supplement: Supplementary file 1 — Supplementary Information. [file 41598_2022_5594_MOESM1_ESM.pdf]

Table S1 Collected Rockburst database

No	Rock type	σ_θ / Mpa	σ_c / Mpa	σ_t / MPa	σ_θ/σ_c	σ_c/σ_t	W_{et}	Rockburst intensity	Reference
1	Granodiorite	90.00	170	11.30	0.53	15.04	9	Moderate	Zhou et al. [1]
2	Syenite	90.00	220	7.40	0.41	29.73	7.3	Light	
3	Granodiorite	62.60	165	9.40	0.38	17.55	9	Light	
4	Granite	55.40	176	7.30	0.31	24.11	9.3	Moderate	
5	Dolomitic Limestone	30.00	88.7	3.70	0.34	23.97	6.6	Moderate	
6	Granite	48.75	180	8.30	0.27	21.69	5	Moderate	
7	Quartzite	80.00	180	6.70	0.44	26.87	5.5	Light	
8	Quartz Diorite	89.00	236	8.30	0.38	28.43	5	Moderate	
9	Marble	98.60	120	6.50	0.82	18.46	3.8	Moderate	
10	Lead and Zinc Ore	108.40	140	8.00	0.77	17.50	5	Strong	
11	Ni Nepheline- P nepheline	57.00	180	8.30	0.32	21.69	5	Moderate	
12	Gneissic Granite	50.00	130	6.00	0.38	21.67	5	Moderate	
13	Granitic Gneiss	62.50	175	7.25	0.36	24.14	5	Moderate	
14	Granite	75.00	180	8.30	0.42	21.69	5	Moderate	
15	Biotite angle, Flash Plagioclase Schist	11.00	115	5.00	0.10	23.00	5.7	None	
16	Diorite Granite	43.40	123	6.00	0.35	20.50	5	Moderate	
17	Granite	18.80	178	5.70	0.11	31.23	7.4	None	
18	Limestone	34.00	150	5.40	0.23	27.78	7.8	None	
19	Granite	56.10	131.99	9.44	0.43	13.98	7.44	Moderate	
20	Granite	54.20	134	9.10	0.40	0.15	7.1	Moderate	
21	Granite	70.30	128.3	8.70	0.55	0.15	6.4	Moderate	
22	Granite	60.70	111.5	7.86	0.54	14.19	6.16	Strong	

23	Migmatite	54.20	134	9.09	0.40	15.00	7.08	Moderate
24	Migmatite	70.30	129	8.73	0.55	11.40	6.43	Moderate
25	Biotite granite porphyry	91.23	157.63	11.96	0.58	13.18	6.27	Strong
26	Biotite granite porphyry	66.77	148.48	8.47	0.45	17.53	5.08	Light
27	Biotite granite porphyry	51.50	132.05	6.33	0.39	20.86	4.63	Moderate
28	Biotite granite porphyry	35.82	127.93	4.43	0.28	28.90	3.67	Light
29	Biotite limestone	21.50	107.52	2.98	0.20	36.04	2.29	None
30	Biotite limestone	18.32	96.41	2.01	0.19	47.93	1.87	None
31	Biotite limestone	110.30	167.19	12.67	0.66	13.20	6.83	Strong
32	Biotite limestone	26.06	118.46	3.51	0.22	33.75	2.89	Light
33	Biotite granite porphyry	16.62	156.86	10.66	0.11	14.71	4.83	Moderate
34	Biotite granite porphyry	16.47	156.9	10.33	0.11	15.19	4.39	Moderate
35	Biotite granite porphyry	16.43	157.95	11.06	0.10	14.28	4.99	Strong
36	Biotite granite porphyry	16.30	155.28	10.63	0.11	14.61	4.4	Moderate
37	Biotite granite porphyry	15.97	114.07	11.96	0.14	9.54	2.4	None
38	Biotite granite porphyry	19.14	106.31	11.96	0.18	8.89	2.07	None
39	Biotite granite porphyry	12.96	117.81	11.96	0.11	9.85	2.49	None
40	Biotite granite porphyry	31.05	147.85	11.96	0.21	12.36	3	Moderate
41	Biotite granite porphyry	29.09	138.5	11.96	0.21	11.58	2.77	None
42	Biotite granite porphyry	32.40	140.88	11.96	0.23	11.78	2.86	Light
43	Biotite granite porphyry	34.89	151.7	10.66	0.23	14.23	3.17	Light
44	Biotite granite porphyry	16.21	135.07	10.33	0.12	13.08	2.49	Light
45	Biotite granite porphyry	30.56	160.83	11.06	0.19	14.54	3.63	Strong
46	Biotite granite porphyry	19.36	113.87	4.43	0.17	25.70	2.38	Light
47	Biotite limestone	33.15	106.94	2.98	0.31	35.89	2.15	Moderate
48	Biotite limestone	9.74	88.51	2.98	0.11	29.70	1.77	None

49	Biotite limestone	11.75	83.96	2.98	0.14	28.17	2.15	None
50	Biotite limestone	39.94	117.48	2.98	0.34	39.42	2.37	Light
51	Biotite limestone	39.82	128.46	2.98	0.31	43.11	2.4	Moderate
52	Biotite limestone	46.22	140.07	2.01	0.33	69.69	3.29	Light
53	Biotite limestone	30.95	123.79	12.67	0.25	9.77	2.57	Light
54	Biotite limestone	40.99	186.3	12.67	0.22	14.70	4.1	Moderate
55	Biotite limestone	20.82	122.47	12.67	0.17	9.67	2.81	Light
56	Biotite limestone	36.09	164.05	12.67	0.22	12.95	3.59	Moderate
57	Clayey sandstone	7.28	52	3.70	0.14	14.05	1.3	None
58	Marble	9.57	99.7	4.80	0.10	20.77	3.8	None
59	Sandstone	34.15	54.2	12.10	0.63	4.48	3.17	Light
60	Granite	60.00	135	15.04	0.44	8.98	4.86	Light
61	Marble	60.00	66.49	9.72	0.90	6.84	2.15	Light
62	Migmatite	60.00	106.38	11.20	0.56	9.50	6.11	Light
63	Peridotite	60.00	86.03	7.14	0.70	12.05	2.85	Light
64	Lherzolite	60.00	149.19	9.30	0.40	16.04	3.5	Light
65	Amphibolite	60.00	136.79	10.42	0.44	13.13	2.12	Light
66	Sandstone	63.80	110	4.50	0.58	24.40	6.31	Moderate
67	Dolomite	2.60	20	3.00	0.13	6.67	1.39	None
68	Phosphate rock	44.40	120	5.00	0.37	24.00	5.1	Light
69	Red Shale	13.50	30	2.67	0.45	11.20	2.03	Light
70	Sandstone	70.40	110	4.50	0.64	24.40	6.31	Moderate
71	Dolomite	3.80	20	3.00	0.19	6.67	1.39	None
72	Phosphate rock	57.60	120	5.00	0.48	24.00	5.1	Moderate
73	Red Shale	19.50	30	2.67	0.65	11.20	2.03	Moderate
74	Sandstone	81.40	110	4.50	0.74	24.40	6.31	Strong

75	Dolomite	4.60	20	3.00	0.23	6.67	1.39	None
76	Phosphate rock	73.20	120	5.00	0.61	24.00	5.1	Moderate
77	Red Shale	30.00	30	2.67	1.00	11.20	2.03	Strong
78	Limestone	15.20	53.8	5.56	0.28	9.68	1.92	None
79	Diorite	88.90	142	13.20	0.63	10.70	3.62	Strong
80	Iron ore	59.82	85.8	7.31	0.70	11.70	2.78	Moderate
81	Skarn	32.30	67.4	6.70	0.48	10.10	1.1	None
82	Dolomitic limestone	30.10	88.7	3.70	0.34	23.97	6.6	Strong
83	Granite	18.80	171.5	6.30	0.11	27.22	7	None
84	Limestone	34.00	149	5.90	0.23	25.25	7.6	Light
85	Clay sandstone	38.20	53	3.90	0.72	13.59	1.6	None
86	Marble	11.30	90	4.80	0.13	18.75	3.6	None
87	Limestone	92.00	263	10.70	0.35	24.58	8	Light
88	Diorite	62.40	235	9.50	0.27	24.74	9	Strong
89	Granite	43.40	136.5	7.20	0.32	18.96	5.6	Strong
90	Diastatite anorthose	11.00	105	4.90	0.10	21.43	4.7	None
91	Mica Marble	46.40	100	4.90	0.46	20.40	2	Light
92	Grey-white Marble	23.00	80	3.00	0.29	26.80	0.85	Light
93	Granophyric Marble	46.20	105	5.30	0.44	19.70	2.3	Light
94	Crystal Tuff	35.00	133.4	9.30	0.26	14.34	2.9	Light
95	N/A	13.90	124	4.22	0.11	29.40	2.04	None
96	N/A	17.40	161	3.98	0.14	31.40	2.19	Light
97	N/A	19.00	153	4.48	0.15	28.10	2.11	Light
98	N/A	19.70	142	4.55	0.16	27.90	2.26	Light
99	Marble	18.70	81.2	10.60	0.23	7.66	1.5	None
100	Marble	23.60	82.8	11.20	0.29	7.39	1.5	None

101	Granite Porphyry	28.60	123.6	11.50	0.23	10.75	2.5	None
102	Granite Porphyry	72.00	120.5	14.90	0.60	8.09	2.5	None
103	Diorite	29.80	132.2	7.80	0.23	16.95	4.6	None
104	Diorite	44.60	130.5	11.09	0.34	11.77	4.6	None
105	Diorite	66.10	135.2	10.90	0.49	12.40	4.6	Light
106	Diorite	99.40	129.5	11.30	0.77	11.46	4.6	Light
107	Dioritic Porphyrite	33.60	156.3	10.20	0.21	15.32	5.2	Light
108	Dioritic Porphyrite	109.50	155.8	11.77	0.70	13.24	5.2	Moderate
109	Magnetite	26.90	92.6	9.52	0.29	9.73	3.7	Light
110	Magnetite	38.30	90.1	10.20	0.43	8.83	3.7	Moderate
111	Magnetite	83.90	95.6	8.69	0.88	11.00	3.7	Light
112	Granite	55.90	126.8	6.56	0.44	19.33	8.1	Strong
113	Granite	109.90	128.5	9.63	0.86	13.34	8.1	Strong
114	Skarn	59.90	96.5	8.00	0.62	12.06	1.8	Light
115	Quartz- feldspar Porphyry	68.00	106.8	6.10	0.64	17.51	7.2	Strong
116	Limestone	50.60	63.83	5.06	0.79	12.61	2.23	Light
117	Limestone	50.60	85.36	4.91	0.59	17.38	3.41	Light
118	Lead-zinc	50.60	104.97	6.18	0.48	16.99	10.9	Strong
119	Pyrite	50.60	153.1	10.48	0.33	14.61	3.14	Light
120	Gneissic granite	120.80	151.6	10.10	0.80	15.01	20	Strong
121	Porphyritic biotite granite	119.32	138.6	7.74	0.86	17.91	30	Strong
122	Porphyritic granite	95.67	127.37	10.51	0.75	12.12	30	Strong
123	Monzogranite	114.44	174.71	14.42	0.66	12.12	10	Strong
124	Monzogranite	127.60	145.42	13.70	0.88	10.61	10	Strong
125	Monzogranite	126.41	158.03	14.32	0.80	11.04	10	Strong
126	Monzogranite	108.53	113.37	10.43	0.96	10.87	10	Strong

127	Metasandston e	29.04	124.15	5.00	0.23	24.30	4.39	None
128	Sandy slate	40.87	139	6.00	0.29	23.17	0.81	None
129	Metasandstone	50.09	124	5.00	0.40	24.80	6.53	Light
130	Sandy slate	59.09	88.25	3.60	0.67	24.51	6.14	Moderate
131	Metasandstone	62.13	124	5.00	0.50	24.80	4.62	Light
132	Sandy slate	40.90	88.25	3.60	0.46	24.51	4.61	Light
133	Sandy slate	22.93	88.25	3.60	0.26	24.51	0.81	None
134	Amphibolite plagiogneiss	47.50	86.3	15.60	0.55	5.53	6.3	Moderate
135	Black mica oblique gneiss	47.50	61.1	5.30	0.78	11.53	7.2	Moderate
136	Copper ore	47.50	99.2	7.30	0.48	13.59	8.31	Moderate
137	Diabase	47.50	91.3	14.50	0.52	6.30	21	Moderate
138	Amphibolite plagiogneiss	67.20	86.3	15.60	0.78	5.53	6.3	Moderate
139	Black mica oblique gneiss	67.20	61.1	5.30	1.10	11.53	7.2	Moderate
140	Copper ore	67.20	99.2	7.30	0.68	13.59	8.31	Moderate
141	Diabase	67.20	91.3	14.50	0.74	6.30	21	Moderate
142	Amphibolite plagiogneiss	77.00	86.3	15.60	0.89	5.53	6.3	Strong
143	Black mica oblique gneiss	77.00	61.1	5.30	1.26	11.53	7.2	Strong
144	Copper ore	77.00	99.2	7.30	0.78	13.59	8.31	Strong
145	Diabase	77.00	91.3	14.50	0.84	6.30	21	Strong
146	Amphibolite plagiogneiss	225.50	86.3	15.60	2.61	5.53	6.3	Strong
147	Black mica oblique gneiss	225.50	61.1	5.30	3.69	11.53	7.2	Strong
148	Copper ore	225.50	99.2	7.30	2.27	13.59	8.31	Strong
149	Diabase	225.50	91.3	14.50	2.47	6.30	21	Strong
150	Amphibolite plagiogneiss	274.30	86.3	15.60	3.18	5.53	6.3	Strong
151	Black mica oblique gneiss	274.30	61.1	5.30	4.49	11.53	7.2	Strong
152	Copper ore	274.30	99.2	7.30	2.77	13.59	8.31	Strong

153	Diabase	274.30	91.3	14.50	3.00	6.30	21	Strong
154	Amphibolite plagiogneiss	297.80	86.3	15.60	3.45	5.53	6.3	Strong
155	Black mica oblique gneiss	297.80	61.1	5.30	4.87	11.53	7.2	Strong
156	Copper ore	297.80	99.2	7.30	3.00	13.59	8.31	Strong
157	Diabase	297.80	91.3	14.50	3.26	6.30	21	Strong
158	Huanglong group marble	77.69	74.04	8.96	1.05	8.26	1.33	None
159	Qixia group marble	77.07	78.3	6.80	0.98	11.51	3.11	Moderate
160	Skarn	67.18	132.2	16.40	0.51	8.06	3.97	Moderate
161	Garnet skarn	75.03	128.6	13.00	0.58	9.89	5.76	Moderate
162	Quartz sandstone	80.54	237.2	17.66	0.34	13.43	6.38	Moderate
163	Siltstone	80.04	171.3	22.60	0.47	7.58	7.27	Strong
164	Diorite porphyry	72.56	304.2	20.90	0.24	14.56	10.57	Strong
165	Limestone	47.56	58.5	3.50	0.81	16.71	5	Light
166	Limestone	43.62	78.1	3.20	0.56	24.41	6	Light
167	Limestone	47.56	80.3	3.50	0.59	22.94	5	Moderate
168	Limestone	44.71	82.4	4.70	0.54	17.53	6.6	Moderate
169	Rhyolite	25.70	59.7	1.30	0.43	45.90	1.7	None
170	Rhyolite	26.90	62.8	2.10	0.42	29.90	2.4	Light
171	Rhyolite	40.40	72.1	2.10	0.56	34.30	1.9	Light
172	Rhyolite	39.40	65.2	2.30	0.60	28.30	3.4	Moderate
173	Rhyolite	38.20	71.4	3.40	0.53	21.00	3.6	Moderate
174	Rhyolite	45.70	69.1	3.20	0.66	21.50	4.1	Moderate
175	Rhyolite	35.80	67.8	3.80	0.52	17.80	4.3	Moderate
176	Rhyolite	39.40	69.2	2.70	0.57	25.60	3.8	Moderate
177	Rhyolite	40.60	66.6	2.60	0.61	25.60	3.7	Moderate
178	Rhyolite	39.00	70.1	2.40	0.56	29.20	4.8	Moderate

179	Rhyolite	57.20	80.6	2.50	0.71	32.20	5.5	Strong
180	Rhyolite	55.60	114	2.30	0.49	49.50	4.7	Moderate
181	Rhyolite	56.90	123	2.70	0.46	45.50	5.2	Moderate
182	Rhyolite	62.10	132	2.40	0.47	55.00	5	Moderate
183	Rhyolite	29.70	116	2.70	0.26	42.90	3.7	Light
184	Rhyolite	29.10	94	2.60	0.31	36.10	3.2	Light
185	Rhyolite	27.80	90	2.10	0.31	42.80	1.8	None
186	Rhyolite	30.30	88	3.10	0.34	28.30	3	Light
187	Rhyolite	55.60	114	2.30	0.49	49.50	4.7	Moderate
188	Rhyolite	41.60	67.6	2.70	0.61	25.00	3.7	Moderate
189	Rhyolite	40.10	72.1	2.30	0.55	31.30	4.6	Moderate
190	Rhyolite	58.20	83.6	2.60	0.69	32.10	5.9	Strong
191	Rhyolite	56.80	112	2.20	0.50	50.90	5.2	Moderate
192	N/A	47.56	58.5	3.50	0.81	16.71	5	Light
193	Limestone and shale	42.40	50	6.10	0.85	8.20	5.3	Light
194	Limestone and shale	63.60	50	4.00	1.27	12.50	5.3	Moderate
195	Sandstone Slate	18.64	70	4.85	0.27	14.43	7.27	Strong
196	Sandstone Slate	18.20	60	1.90	0.30	31.58	2.84	Light
197	Granite	53.00	147.2	7.18	0.36	20.50	5	Moderate
198	Bedrock	16.40	156.23	10.51	0.10	14.86	4.14	Light
199	Bedrock	16.40	156.14	10.30	0.11	15.16	4.04	Light
200	Bedrock	16.40	157.34	10.55	0.10	14.91	4.26	Moderate
201	Bedrock	16.40	155.63	10.42	0.11	14.94	4.2	Moderate
202	Dolomitic limestone	12.00	30	5.58	0.40	5.38	5.1	None
203	Biotite granite	50.28	77.3	7.65	0.65	10.11	2.47	Light
204	Phyllic of granite	50.28	94.7	5.26	0.53	18.01	2.96	Moderate

205	phyllic of granitic cataclasite	50.28	59	5.23	0.85	11.28	0.88	None
206	Biotite granite	44.80	77.3	7.65	0.58	10.11	2.47	Light
207	Biotite granite	48.00	77.3	7.65	0.62	10.11	2.47	Light
208	Biotite granite	53.40	77.3	7.65	0.69	10.11	2.47	Light
209	Biotite granite	54.90	77.3	7.65	0.71	10.11	2.47	Light
210	Phyllic of granite	44.80	94.7	5.26	0.47	18.01	2.96	Moderate
211	Phyllic of granite	48.00	94.7	5.26	0.51	18.01	2.96	Moderate
212	Phyllic of granite	53.40	94.7	5.26	0.56	18.01	2.96	Moderate
213	Phyllic of granite	54.90	94.7	5.26	0.58	18.01	2.96	Moderate
214	Phyllic of granitic cataclasite	44.80	59	5.23	0.76	11.28	0.88	None
215	Phyllic of granitic cataclasite	48.00	59	5.23	0.81	11.28	0.88	None
216	Phyllic of granitic cataclasite	53.40	59	5.23	0.91	11.28	0.88	None
217	Phyllic of granitic cataclasite	54.90	59	5.23	0.93	11.28	0.88	None
218	Sandstone	12.00	85	3.60	0.14	23.61	1.5	None
219	Marble sandstone	21.00	103	4.10	0.20	25.12	2.4	Light
220	Marble sandstone	28.00	100	3.90	0.28	25.64	2.3	Light
221	Brecciated marble	47.00	122	5.50	0.39	22.18	3.4	Light
222	Marble	52.00	117	4.80	0.44	24.38	3.2	Light
223	Marble	42.00	117	4.80	0.36	24.38	3.2	Light
224	Marble	32.00	117	4.80	0.27	24.38	3.2	Light

225	Banded marble argillaceous limestone	24.00	110	4.40	0.22	25.00	3	Light
226	Argillaceous limestone	20.00	112	4.70	0.18	23.83	2.5	None
227	Weathered and fresh welded tuff, tuff breccia and the K-feldspar granite porphyry	32.80	160	6.60	0.21	24.30	4.6	Light
228	Weathered and fresh welded tuff, tuff breccia and the K-feldspar granite porphyry	44.80	160	6.80	0.28	23.60	4.9	Light
229	Weathered and fresh welded tuff, tuff breccia and the K-feldspar granite porphyry	50.90	160	7.50	0.32	21.30	5.3	Moderate
230	Weathered and fresh welded tuff, tuff breccia and the K-feldspar granite porphyry	44.80	160	6.70	0.28	23.80	4.8	Light
231	Weathered and fresh welded tuff, tuff breccia and the K-feldspar granite porphyry	22.40	160	6.60	0.14	24.10	4.3	Light
232	Amphibolite syenite	20.61	54.23	21.49	0.38	2.52	3.17	Light
233	Granite	48.00	120	1.50	0.40	80.00	5.8	Moderate
234	Granite	49.50	110	1.50	0.45	73.33	5.7	Moderate
235	Granite	63.00	115	1.50	0.55	76.67	5.7	Moderate
236	Dolomitic limestone	17.39	102.3	1.30	0.17	78.69	6.58	Moderate
237	Dolomitic limestone	17.02	85.09	1.30	0.20	65.45	6.14	Moderate
238	Dolomite	16.70	83.5	1.30	0.20	64.23	6.53	Moderate
239	Dolomite	17.35	86.77	1.30	0.20	66.75	3.22	Moderate

240	Sandy slate	16.87	80.33	1.30	0.21	61.79	6.92	Moderate	
241	Sandy slate	17.08	94.9	1.30	0.18	73.00	6.91	Moderate	
242	Moderately weathered granodiorite porphyry	61.72	92.4	8.28	0.67	11.16	5.43	Light	
243	Fresh granodiorite porphyry	126.72	189.7	8.95	0.67	21.20	5.43	Light	
244	Granite porphyry	57.97	125.37	7.74	0.67	21.20	2.86	Light	
245	Granite porphyry	57.97	96.16	3.77	0.46	16.20	2.53	Light	
246	Granite porphyry	57.97	70.68	4.19	0.60	25.51	2.87	Light	
247	Yanshanian biotite granite	98.02	148.52	6.66	0.66	22.30	3.23	Moderate	Xue et al. [2]
248	Yanshanian biotite granite	116.88	162.33	12.30	0.72	13.20	5.23	Strong	
249	Yanshanian biotite granite	43.21	116.78	3.93	0.37	29.73	3.52	Light	
250	Yanshanian biotite granite	45.92	109.33	3.34	0.42	32.77	2.97	Light	
251	Yanshanian biotite granite	27.60	98.56	2.31	0.28	42.73	2.17	None	
252	Yanshanian biotite granite	76.80	156.73	7.79	0.49	20.13	3.82	Moderate	
253	Yanshanian biotite granite	38.12	100.32	3.49	0.38	28.77	3.02	Light	
254	Yanshanian biotite granite	102.38	142.2	5.17	0.72	27.52	4.3	Moderate	
255	Yanshanian biotite granite	110.62	160.32	9.69	0.69	16.55	5.72	Strong	
256	Yanshanian biotite granite	40.99	97.6	6.30	0.42	15.50	3.2	Light	
257	Yanshanian biotite granite	58.12	100.2	3.33	0.58	30.12	4.5	Light	
258	Yanshanian biotite granite	23.39	106.32	2.92	0.22	36.42	1.75	None	
259	Yanshanian biotite granite	81.75	125.77	12.14	0.65	10.36	5.75	Moderate	
260	Yanshanian biotite granite	90.99	146.75	7.58	0.62	19.35	4.5	Moderate	
261	Yanshanian biotite granite	61.42	107.75	3.45	0.57	31.20	3.15	Light	
262	Yanshanian biotite granite	104.49	160.75	13.01	0.65	12.36	5.41	Strong	
263	Yanshanian biotite granite	86.56	146.72	7.83	0.59	18.75	4.2	Moderate	
264	Yanshanian biotite granite	118.77	162.7	5.48	0.73	29.70	3.82	Moderate	

265	Yanshanian biotite granite	35.34	95.5	2.26	0.37	42.30	2.75	None	
266	Yanshanian biotite granite	39.11	105.7	2.83	0.37	37.35	3.08	Light	
267	Kimberlite	18.17	49.1	1.56	0.37	31.40	3.3	Moderate	Pu et al. [3]
268	Kimberlite	21.00	60	3.17	0.35	18.90	1.7	Moderate	
269	Kimberlite	31.16	82	3.87	0.38	21.20	2.3	Moderate	
270	Kimberlite	46.38	74.8	2.98	0.62	25.10	3.2	Moderate	
271	Kimberlite	48.64	76	4.09	0.64	18.60	2.5	Moderate	
272	Kimberlite	22.92	57.3	1.43	0.40	40.00	1.5	Moderate	
273	Kimberlite	99.09	112.6	3.74	0.88	30.10	5.2	Moderate	
274	Kimberlite	35.16	79.9	3.12	0.44	25.60	2.5	Light	
275	Kimberlite	15.84	49.5	2.16	0.32	22.90	2.8	Moderate	
276	Kimberlite	13.02	65.1	2.28	0.20	28.50	1.2	Moderate	
277	Kimberlite	21.12	52.8	2.18	0.40	24.20	2.3	Moderate	
278	Kimberlite	29.12	57.1	3.34	0.51	17.10	2.2	Moderate	
279	N/A	30.9	238	7.60	0.13	31.20	7.40	None	Liu et al. [4]
280	N/A	64.5	215	9.00	0.30	24.00	6.60	Moderate	
281	N/A	75.5	151	18.20	0.50	8.30	3.10	Light	
282	N/A	68.5	185	7.70	0.37	24.10	5.00	Moderate	
283	N/A	75.6	194	8.90	0.39	21.70	5.00	Moderate	
284	N/A	57.9	181	7.50	0.32	24.10	9.30	Moderate	
285	N/A	72.6	173	8.00	0.42	21.70	5.20	Moderate	
286	N/A	54.9	183	9.00	0.30	20.40	5.10	Light	
287	N/A	62.7	196	9.00	0.32	21.70	5.00	Moderate	
288	N/A	24.1	241	0.40	0.10	23.00	5.70	None	
289	N/A	50.5	187	8.60	0.27	21.70	5.00	Moderate	
290	N/A	78.8	179	6.7	0.44	26.7	5.5	Light	

291	N/A	31.2	156	13.9	0.2	11.2	3.6	None	
292	N/A	61.6	162	9.2	0.38	17.6	9	Moderate	
293	N/A	78.1	192	6.5	0.41	29.7	7.3	Light	
294	N/A	132.4	172	9.8	0.77	17.5	5.5	Strong	
295	Fused tuff	14.49	70.21	2.04	0.21	34.42	3.11	Light	Jia et al. [5]
296	Sandstone	17.79	81.8	2.45	0.22	33.39	3.6	Light	
297	Sandstone	22.21	90	3.1	0.25	29.03	4.3	Light	
298	Micro-weathered tuff	25.62	99.6	3.42	0.26	29.12	4.6	Moderate	
299	Micro-weathered tuff	25.92	72.8	2.6	0.36	28.00	4.4	Moderate	
300	Weak-weathered tuff	26.68	46.8	1.8	0.57	26.00	2.7	Moderate	
301	Griotte	30.9	82.56	6.5	0.37	12.70	3.2	Light	Du et al. [6]
302	Siltstone	61	171.5	22.6	0.36	7.59	7.5	Light	
303	Skarn	89	128.6	13.2	0.69	9.74	4.9	Strong	
304	Quartz sandstone	12.3	237.1	17.66	0.05	13.43	6.9	None	
305	Dioritic porphyrite	55.6	256.5	18.9	0.22	13.57	9.1	Strong	
306	Granite	67.2	222.6	10.6	0.30	21.00	7.8	Strong	
307	Syenite	91.3	225.6	17.2	0.40	13.12	7.3	Strong	
308	Middle and upper Triassic marble, limestone, crystalline limestone and sandstone, and slate	67.73	112.5	4.1	0.60	27.44	6.02	Moderate	Wu et al. [7]
309	Middle and upper Triassic marble, limestone, crystalline limestone and sandstone, and slate	64.43	112.5	3.4	0.57	33.09	5.8	Light	

310	Middle and upper Triassic marble, limestone, crystalline limestone and sandstone, and slate	62.88	112.5	3.4	0.56	33.09	5.62	Moderate
311	Middle and upper Triassic marble, limestone, crystalline limestone and sandstone, and slate	54.51	99.59	4.1	0.55	24.29	3.26	Strong
312	Middle and upper Triassic marble, limestone, crystalline limestone and sandstone, and slate	52.08	112.5	3.4	0.46	33.09	4.68	Moderate
313	Middle and upper Triassic marble, limestone, crystalline limestone and sandstone, and slate	53.93	112.5	3.4	0.48	33.09	4.5	Moderate
314	Middle and upper Triassic marble, limestone, crystalline limestone and sandstone, and slate	68.98	112.5	4.1	0.61	27.44	5.32	Moderate

Note: N/A is not available.

Reference

1 Zhou, J., Li, X. & Mitri, H. S. Classification of Rockburst in Underground Projects: Comparison of Ten Supervised Learning Methods. *Journal of Computing in Civil Engineering* **30**, doi:10.1061/(asce)cp.1943-5487.0000553 (2016).

2 Xue, Y. *et al.* Prediction of rock burst in underground caverns based on rough set and extensible comprehensive evaluation. *Bulletin of Engineering Geology and the*

Environment **78**, 417-429 (2019).

3 Pu, Y., Apel, D. B. & Xu, H. Rockburst prediction in kimberlite with unsupervised learning method and support vector classifier. *Tunnelling and Underground Space Technology* **90**, 12-18 (2019).

4 Liu, R., Ye, Y., Hu, N., Chen, H. & Wang, X. Classified prediction model of rockburst using rough sets-normal cloud. *Neural Computing and Applications* **31**, 8185-8193 (2019).

5 Jia, Q., Wu, L., Li, B., Chen, C. & Peng, Y. The comprehensive prediction model of rockburst tendency in tunnel based on optimized unascertained measure theory. *Geotechnical and Geological Engineering* **37**, 3399-3411 (2019).

6 Du Zijian, X. M., Zhenping, L. & Xuan, W. Laboratory integrated evaluation method for engineering wall rock rock-burst [J]. *Gold* **11 (in Chinese)** (2006).

7 Wu, S., Wu, Z. & Zhang, C. Rock burst prediction probability model based on case analysis. *Tunnelling and Underground Space Technology* **93**, 103069 (2019).
